# Pubertal development mediates the association between family environment and brain structure and function in childhood

**DOI:** 10.1017/S0954579419000580

**Published:** 2020-05

**Authors:** Sandra Thijssen, Paul F. Collins, Monica Luciana

**Affiliations:** 1 Department of Psychology, Education, and Child Studies, Erasmus University Rotterdam, the Netherlands; 2 Department of Psychology, University of Minnesota, Minneapolis, MN, USA

**Keywords:** accelerated development, amygdala–medial prefrontal cortex circuit, family environment, psychosocial acceleration theory, pubertal development

## Abstract

Psychosocial acceleration theory suggests that pubertal maturation is accelerated in response to adversity. In addition, suboptimal caregiving accelerates development of the amygdala–medial prefrontal cortex circuit. These findings may be related. Here, we assess whether associations between family environment and measures of the amygdala–medial prefrontal cortex circuit are mediated by pubertal development in more than 2000 9- and 10-year-old children from the Adolescent Brain Cognitive Development Study (http://dx.doi.org/10.15154/1412097). Using structural equation modeling, demographic, child-reported, and parent-reported data on family dynamics were compiled into a higher level family environment latent variable. Magnetic resonance imaging preprocessing and compilations were performed by the Adolescent Brain Cognitive Development Study's data analysis core. Anterior cingulate cortex (ACC) thickness, area, white matter fractional anisotropy, amygdala volume, and cingulo-opercular network–amygdala resting-state functional connectivity were assessed. For ACC cortical thickness and ACC fractional anisotropy, significant indirect effects indicated that a stressful family environment relates to more advanced pubertal stage and more mature brain structure. For cingulo-opercular network–amygdala functional connectivity, results indicated a trend in the expected direction. For ACC area, evidence for quadratic mediation by pubertal stage was found. Sex-stratified analyses suggest stronger results for girls. Despite small effect sizes, structural measures of circuits important for emotional behavior are associated with family environment and show initial evidence of accelerated pubertal development.

During childhood and to a lesser extent during adolescence, the quality of parenting and the larger family environment exert a considerable effect on several domains of child development. It is not surprising then that suboptimal environments are associated with poor outcomes. For example, parental separation and parental or parent–child conflict have been associated with child behavior problems and poor school performance (e.g., Erman & Härkönen, 2017; Harold, Aitken, & Shelton, 2007; Kreidl, Štípková, & Hubatková, 2017; Moed et al., 2017). Associations with physical development have also been reported, suggesting that children experiencing family adversity may have an earlier onset of puberty (e.g., Ellis & Garber, 2000; Jorm, Christensen, Rodgers, Jacomb, & Easteal, 2004; Moffitt, Caspi, Belsky, & Silva, 1992). Caregiving also affects brain development in ways that are only beginning to be understood. In a typically developing, nonclinical sample, lower levels of early parental sensitivity were associated with smaller total brain and gray matter volume (controlled for infant head size) later in childhood (Kok et al., 2015). In a different study, maternal sensitivity toward the child at age 12 predicted reduced growth in the amygdala and greater thinning of the orbitofrontal cortex 4 years later (Whittle et al., 2014). Similarly to pubertal development, there is some evidence that brain development, specifically the functional development of the amygdala–medial prefrontal cortex (mPFC) circuit, may be accelerated in the context of lower levels of supportive parental care (Gee, Gabard-Durnam, et al., 2013; Thijssen et al., 2017). It has been hypothesized that this acceleration may, in turn, impact the child's emotional functioning. However, it is unclear how these findings relate to the reports of accelerated pubertal development. Here we aim to examine whether associations between a child's family environment and measures of the amygdala–mPFC circuit are mediated by child pubertal stage in a large sample of 9- to 10-year-old children characterized by a broad range of pubertal development.

Accelerated brain development in response to adversity has been reported for the amygdala–mPFC circuit. The first of this circuit's subparts, the amygdala, is suggested to play a role in emotional learning and may facilitate attention to salient cues (Phelps & LeDoux, 2005). The mPFC, in contrast, including the anterior cingulate cortex (ACC), has been implicated in higher order emotional and cognitive functioning (Forbes & Grafman, 2010; Ridderinkhof, Ullsperger, Crone, & Nieuwenhuis, 2004), and may provide top-down regulation of amygdala reactivity to emotional stimuli. Functional coupling of the amygdala and mPFC may thus be involved in emotion regulation (Hariri, Mattay, Tessitore, Fera, & Weinberger, 2003; Ochsner, Bunge, Gross, & Gabrieli, 2002; Phan et al., 2005). In children, responses of the amygdala and mPFC to emotional stimuli (i.e., fearful faces) are positively correlated (Gee, Humphreys, et al., 2013). In contrast, adolescents and adults show a negative pattern of amygdala–mPFC functional connectivity in response to the same stimuli, which is interpreted as effective top-down control of the amygdala by the mPFC (however, as findings are correlational, the opposite pattern of the amygdala decreasing mPFC activity could also be true). In childhood, emotion regulation skills are still developing, and parents play an important role in helping their children regulate their emotions (Tottenham, 2015). When a photo of the mother (vs. a stranger) is presented during a functional magnetic resonance imaging (fMRI) task, children show evidence of maternal buffering and, similar to adolescents and adults, display negative connectivity between the amygdala and the mPFC when processing fearful faces (Gee et al., 2014). Of importance, previously institutionalized youth have been shown to demonstrate negative connectivity earlier in development and without presentation of parental stimuli, suggesting accelerated development in response to extreme early life adversity (Gee, Gabard-Durnam, et al., 2013). Following up on this finding, Thijssen et al. (2017) showed that, in typically developing 6- to 10-year-old children, amygdala–mPFC connectivity at rest increased with age in children with less sensitive parents, but it did not in children with more sensitive parents. As amygdala–mPFC resting-state functional connectivity has been found to increase from age 10.5 onward (Gabard-Durnam et al., 2014), these results provide initial evidence for accelerated development of the amygdala–mPFC circuit in response to normative, yet less than optimal, caregiving.

These findings of accelerated maturation in response to caregiving adversity are in line with psychosocial acceleration theory (Belsky, Steinberg, & Draper, 1991). This theory poses that children adaptively adjust their development to match local conditions. Parental care and investment provide children with information about availability and predictability of resources and relationships, with less than optimal care suggesting scarcity of resources and low quality of interpersonal relationships. According to this theory, children may respond to adversity with a speeding up of development, as this may ultimately increase their reproductive opportunities and success. Several studies have shown that parental warmth is associated with delayed puberty in girls (defined as later menarche, earlier Tanner stage, or earlier pubertal stage according to the Pubertal Development Scale; Ellis, McFadyen-Ketchum, Dodge, Pettit, & Bates, 1999; Graberc, Brooks-Gunn, & Warren, 1995; Romans, Martin, Gendall, & Herbison, 2003), and that family conflict or parental psychopathology is associated with the earlier initiation of puberty (e.g., Ellis & Garber, 2000; Jorm et al., 2004; Moffitt et al., 1992). Similarly, accelerated development of the amygdala–mPFC circuit may have implications for child fitness and survival. Although a long childhood is critical for the development of highly competent behavior and long-term well-being, in the short run, it may be adaptive to be capable of self-regulation instead of relying on a parent for emotion regulation when quality of parental care is low, even at the cost of long-term health or well-being (Belsky, Ruttle, Boyce, Armstrong, & Essex, 2015; Hochberg & Belsky, 2013).

It is currently unclear what mechanisms may explain the accelerated development of the amygdala–mPFC circuit. It may be that a suboptimal family environment constitutes a source of chronic stress. This stress response may directly affect amygdala–mPFC circuit development (Gee, Gabard-Durnam, et al., 2013). Alternatively, given that an adverse family background has also been associated with precocious pubertal development, it is possible that the precocious emergence of the adolescent/adult pattern of amygdala–mPFC connectivity is a consequence of an early onset of puberty. Several studies have shown correlations between pubertal hormones and brain development and thus provide some evidence of organizing effects of adrenal and gonadal hormones. While sex-dimorphic effects are prominent (Bramen et al., 2011), studies have also reported strong similarities between associations of estradiol and testosterone and adolescent brain development (Herting, Gautam, Spielberg, & Dahl, 2015). Similarly, pubertal developmental stage has been related to brain development in both sexes (Goddings et al., 2014; Herting et al., 2015). In some studies, previously institutionalized children have been reported to experience an earlier onset of puberty (Adolfsson & Westphal, 1981; Proos, 2009; Teilmann, Pedersen, Skakkebaek, & Jensen, 2006), which may relate to the observation of accelerated amygdala–mPFC development in this group. However, several more recent studies reported nonsignificant associations between previous institutionalization and earlier age of menarche (Johnson et al., 2018; Reid et al., 2017). Thus, the literature is inconsistent, which could be due to methodological distinctions across studies.

Although evidence has been found for accelerated *functional* development of the amygdala–mPFC circuit (Gee, Gabard-Durnam, et al., 2013; Thijssen et al., 2017), it is also possible that the development of amygdala and mPFC *structure* is accelerated in response to family adversity or lower quality care. It has been suggested that developmental changes in connectivity may be the consequence of changes in structural connections between two brain regions (Gee, Gabard-Durnam, et al., 2013; Wendelken et al., 2017). Similarly, although not perfectly, brain function and gray matter structure have been found to correlate (Lu et al., 2009), and accelerated development of one modality may suggest acceleration in others. In line with this hypothesis, Tyborowska et al. (2018) report increased developmental gray matter reductions from age 14 to 17 in several brain regions such as the amygdala, insula, and prefrontal cortex in relation to early life stress.

The present study used data from the Adolescent Brain and Cognitive Development (ABCD) Study, an epidemiologically informed sample of 9- to 10-year-old children, to assess the association between family environment and child structural (T1 and diffusion tensor imaging [DTI] data) and functional (resting-state fMRI) brain measures related to the amygdala–mPFC circuit made available by ABCD in the context of the project's first public data release (see Methods). We further examined whether such associations are mediated by pubertal development. We hypothesize that associations between more stressful family environments and child brain structure and function are mediated by accelerated pubertal development. As a precocious onset of puberty in response to a suboptimal family environment has been reported mostly for girls (Ellis, 2004), and as exploratory analyses by Thijssen et al. (2017) suggest that accelerated development of the amygdala–mPFC circuit may be more prominent in girls than in boys, we also performed exploratory analyses stratified by sex.

## Method

### Participants

The present study used data collected for the ABCD Study (first public data release: http://dx.doi.org/10.15154/1412097, downloaded on February 15, 2017; for resting-state fMRI data, data from release 1.1 was used: http://dx.doi.org/10.15154/1412097). The ABCD Study aims to follow a population-based, prospective cohort from ages 9 to 10 years and onward. Data are collected across 21 sites in the United States. Up until the first data release, 4,524 participants were recruited and tested. For information on ABCD recruitment, please see Garavan et al. (2018). The ABCD Study includes a representative sample of both twin and nontwin participants as well as siblings within families. For the present study, 430 twin pairs were excluded, and from each of 83 sibling pairs, 1 child was randomly excluded from the analysis. From an additional 28 sibling pairs, 1 sibling had unusable resting-state fMRI data, and this individual was excluded (see Quality Assurance). We further excluded 194 participants who attended the research center supervised by someone other than their biological parent, in order to increase the validity of the parent reported measures. Finally, 176 children were excluded due to MRI incidental findings. These exclusions resulted in a final sample of 3,183 children. For these children, parents provided informed consent, and children provided assent to participate. Data collection for the ABCD Study was approved by a centralized internal review board of the University of San Diego, as well as by the review boards of all research sites.

### Measures

#### Family environment

For a detailed description and rationale for the measures collected in the ABCD Study that may be relevant for the family environment construct, please see Barch et al. (2017) for demographic, physical and mental health assessments, and Zucker et al. (2018) for the assessment of culture and environment.

In order to create a latent measure reflecting the quality of family environment, three types of information were used: child-reported information about family dynamics and relationships, parent-reported information about family dynamics and relationships, and demographic and parent information. Child-reported questionnaires included an abbreviated version of the maternal acceptance scale of the Child Report of Parent Behavior Inventory (Schaefer, 1965), the conflict scale from the Family Environment Scale (FES; Moos & Moos, 1976), and the Parental Monitoring Survey (Chilcoat & Anthony, 1996). The abbreviated version of the maternal acceptance scale of the Child Report of Parent Behavior Inventory assesses maternal acceptance versus rejection and consists of 5 items (e.g., “smiles at me very often”) measured on a 3-point Likert scale ranging from *not like her* to *a lot like her*. The Parental Monitoring Survey consists of 5 items answered on a 5-point Likert scale ranging from *never* to *always or almost always* (e.g., “how often do your parents know where you are?”), and assesses whether the child believes that his/her parent knows of his/her whereabouts and activities. The conflict scale of the FES consists of 9 true or false items that aim to measure conflict within the family (e.g., “we fight a lot in our family”). The parent-reported information also included the conflict scale from the FES, and was complimented by one background item from the Kiddie Schedule for Affective Disorders and Schizophrenia (Kaufman et al., 1997) measuring the relationship between the parent and the child (“in general, how do you and your child get along?” using a 3-point Likert scale ranging from *very well* to *a lot of conflict*). Demographic and parent information included family yearly income (rated on a 10-point scale ranging from *less than $5,000* to *$200,000 or more*), parental education (6-point scale ranging from *finished high school or less* to *professional school or doctoral degree*; PhenX; Stover, Harlan, Hammond, Hendershot, & Hamilton, 2010), parental psychopathology (total problem score of the Achenbach System of Empirically Based Assessment Adult Self-Report; Achenbach & Rescorla, 2003), parental relationship status (i.e., are biological parents still together?), and the planned nature of the pregnancy (i.e., did parents plan this pregnancy; Kessler et al., 2009).

Using Mplus (Muthén & Muthén, 2007), all questionnaire items were submitted to structural equation modeling (except for the income and parental psychopathology variables, all variables were classified as categorical). We excluded variables with factor loadings <0.2. For the child-reported items, items were first loaded on a latent variable referring to the questionnaire scale. These questionnaire scale latent variables were then combined in a child-reported latent variable. All parent-reported items (9 FES and 1 Kiddie Schedule for Affective Disorders and Schizophrenia items) were combined in a parent-reported latent variable. However, 2 items of the FES showed a low factor loading (<0.2) on the parent variable and were removed from the model. The variables referring to demographical information were combined into a demographics latent variable. The child-reported (STD standardized loading = 0.53), parent-reported (STD standardized loading = 0.55), and demographical latent variables (STD standardized loading = 0.44) were then combined to yield an overall family environment latent variable (see Supplemental Figure S.1 for all factor loadings). The model had reasonable fit, root mean square error of approximation = .040, 95% confidence interval (CI) [.038, .041], comparative fit index = .87, and Tucker–Lewis index = .86. Low scores on the family environment variable indicate a more stressful/less supportive family environment (i.e., increased family conflict, lower parental acceptance and monitoring, lower socioeconomic status, and/or higher parental psychopathology).

Although the child- and parent-reported measures of family dynamics reflect concurrent and not early life relationships, parent–child relationship patterns are relatively stable over time (Loeber et al., 2000), as is socioeconomic status. The demographic latent variable includes a broader time frame of events (planned nature of pregnancy and parental separation during child's life).

#### Child behavior

In order to assess the predictive validity of the family environment variable, family environment was associated with measures of child behavior. From the multitude of child behavior measures available (please see Barch et al., 2017; Zucker et al., 2018, for the conclusive list), we included measures of both positive behavior and behavioral problems, reported by both the parent and the child. As such, we included the broadbent internalizing and externalizing scales of the Child Behavior Checklist (Achenbach & Edelbrook, 1983), and the parent- and child-reported prosocial scale of the Strengths and Difficulties Questionnaire (Goodman, Meltzer, & Bailey, 1998). The items comprising the internalizing (*M* = 5.22, *SD* = 5.44) and externalizing (*M* = 4.25, *SD* = 5.45) scales can be answered on a 3-point Likert scale. The prosocial scale consists of five items that are answered on a 3-point Likert scale (1 = *not true*, 2 = *somewhat true*, 3 = *certainly true*). Three of the five items were retained in the ABCD Study (*M* = 1.75, *SD* = 0.40; *M* = 1.69, *SD* = 0.36, for parent and child report, respectively).

The child behavior measures were nonnormally distributed, with many parents reporting few behavioral problems and many parents and children reporting high prosocial behavior. Thus, the Child Behavior Checklist scales were log transformed, and prosocial behavior scales were inverse transformed.

#### Pubertal stage

Both the child and the parent reported on the child's pubertal stage using the Pubertal Development Scale (Petersen, Crockett, Richards, & Boxer, 1988). Correlations between the parent and child measures were *r* = .562, *p* < .001 for girls and *r* = .197, *p* < .001 for boys, respectively. More parent-reported compared to child-reported pubertal stage scores were available for the sample (3,107 vs. 2,918). Moreover, whereas age correlated significantly with the parent-reported score for both boys and girls (*r* = .096 *p* < .001, *r* = .265 *p* < .001, respectively), as well as with the self-reported score for girls (*r* = .239, *p* < .001), the correlation between age and self-reported pubertal stage for boys was not significant (*r* = –.030, *p* = .218). We, therefore, decided to use the parent-provided data as our primary measure of pubertal stage and supplemented missing scores by child-reported information when available. Although the sample covered the full range of pubertal development, few children were reported to be in Stage 4 (girls: *n* = 34 and boys: *n* = 13) of pubertal development. No parents reported their child to be in pubertal Stage 5, but 4 girls and 4 boys self-reported Stage 5 of pubertal development. Because of these low numbers we combined Stage 3, 4, and 5 as Stage 3+. This recoded variable was used for all analyses, except for the quadratic mediation models. As quadratic mediation models cannot handle categorical mediators, for the quadratic mediation analysis of brain structure, the originally coded variable (pubertal stage) was used and treated continuously. Forty-one girls reported or were reported to have experienced menarche.

#### Magnetic resonance imaging

##### Measures of the amygdala–mPFC circuit

ABCD provides tabulated summary statistics of MRI data based on processing algorithms implemented by its data analytic core (Hagler et al., 2018). Given that both Gee, Gabard-Durnam, et al. (2013) and Thijssen et al. (2017) report correlations between familial environment and amygdala–ACC (which is part of the mPFC) connectivity, we focused on indices of amygdala and ACC gray and white matter structure. As such, we examined amygdala volumes, ACC cortical thickness and area, and ACC white matter fractional anisotropy. Unfortunately, no direct measure of amygdala–mPFC functional connectivity has been released by ABCD to date, but amygdala functional connectivity with several well-characterized resting-state networks (Gordon et al., 2016) was provided in the first data release. Here, we assessed associations between family environment and connectivity of the amygdala and the cingulo-opercular network, which includes the caudal ACC and insula and has been implicated in cognitive control (Dosenbach et al., 2007). This network, however, does not encompass the entire mPFC and includes regions outside of the mPFC. This measure can therefore merely be seen as a proxy of the network of direct interest. Among the available resting-state functional networks that have been analyzed for the ABCD cohort, another network that encompasses a large part of the mPFC is the default mode network. However, as this network also includes regions such as the posterior cingulate cortex, precuneus, and temporoparietal junction, and as its functionality (task negative network involved in self-referencing, theory of mind, and episodic memory; Whitfield-Gabrieli & Ford, 2012) less well matches the function of amygdala–mPFC circuit (emotion regulation), it was not included as an outcome of interest. Comparable to the cingulo-opercular network, the salience network includes the insula and ACC. However, as the salience network only includes a small fraction of the ACC, this network was not selected.

To assess the discriminant validity of our results, we also analyzed measures of motor processing, specifically (mean left and right) precentral cortical thickness and cortical area, white matter fractional anisotropy, as well as somatomotor-mouth–amygdala connectivity. As the motor cortex is one of the first brain regions to reach peak cortical thickness/area (Giedd et al., 1999), we did not expect that family environment would relate to precentral gray matter via pubertal stage.

##### MRI acquisition

For details on MRI acquisition in the ABCD Study, please see Casey et al. (2018). Across all sites, participants were familiarized to the MRI environment using a mock scanner. A 2-hr MRI scanning session was performed on different 3T scanners from Siemens (Siemens Medical Systems, Erlangen, Germany), Philips (Philips Medical Systems, Best, the Netherlands), as well as GE (General Electric, Milwaukee, MI, USA). A T1-weighted anatomical scan was acquired as follows: Siemens scanners: resonance time (TR) = 2500 ms, echo time (TE) = 2.88 ms, flip angle = 8 degrees, 176 transverse slices, voxel size 1 × 1 × 1 mm; Philips scanners: TR = 6.31 ms, TE = 2.9 ms, flip angle = 8 degrees, 255 transverse slices, voxel size 1 × 1 × 1 mm; GE scanners: TR = 2500 ms, TE = 2.0 ms, flip angle = 8 degrees, 208 transverse slices, voxel size 1 × 1 × 1 mm.

Diffusion tensor imaging data were acquired using a multiband, echo-planar imaging sequence with the following parameters: TR = 4100 ms Siemens and GE/5300 ms Philips, TE = 88 ms Siemens/81 ms Philips/89 ms GE, flip angle = 90 degrees Siemens/78 degrees Philips/ 77 degrees GE, matrix = 140 × 140, field of view = 240 mm × 240 mm, slice thickness = 1.7 mm, number of slices = 81, 96 diffusion directions, acquisition time = 7 min 31 s Siemens/9 min 14 s Philips/7 min 30 s GE.

The resting-state fMRI sequence utilized the same gradient-echo blood oxygen level dependent echo-planar imaging sequence for each scanner, with TR = 800 ms, TE = 30 ms, flip angle = 52 degrees, 60 transverse slices, multiband acceleration = 6, and voxel resolution of 2.4 × 2.4 × 2.4 mm. Three to four runs of 5 min were acquired. The first two runs as well as Run 3 and 4 were separated by a 10-s film clip. The second and the third run were separated by a diffusion scan.

##### Preprocessing and MRI data analysis

MRI preprocessing and analyses were performed by the ABCD consortium's data analytic core. Please see Hagler et al. (2018) for detailed information.

T1-weighted images were corrected for gradient nonlinearity distortions (Jovicich et al., 2006). T2-weighted images were registered to T1-weighted images using mutual information (Wells, Viola, Atsumi, Nakajima, & Kikinis, 1996). Intensity nonuniformity correction was performed based on tissue segmentation and sparse spatial smoothing. Data were resampled with 1 mm isotropic voxels into rigid alignment with an atlas brain. Cortical surface reconstruction was performed using FreeSurfer v5.3.0 (https://surfer.nmr.mgh.harvard.edu). Briefly, FreeSurfer was used for skull stripping (Ségonne et al., 2004), white matter segmentation, initial mesh creation (Dale, Fischl, & Sereno, 1999), correction of topological defects (Fischl, Liu, & Dale, 2001; Ségonne, Pacheco, & Fischl, 2007), surface optimization (Dale et al., 1999; Dale & Sereno, 1993; Fischl & Dale, 2000), and nonlinear registration to a spherical surface-based atlas (Fischl, Sereno, Tootell, & Dale, 1999). Subcortical structures were labeled with atlas-based segmentation (Fischl et al., 2002).

As with the T1-weighted images, diffusion images were corrected for gradient nonlinearity distortions (Jovicich et al., 2006). Diffusion data were corrected for eddy current distortion through an iterative model-based approach that used diffusion gradient orientations and amplitudes to predict the pattern of distortions across the entire set of diffusion-weighted volumes in terms of translation, scaling, and shear along the phase-encode direction (Zhuang et al., 2006). During each iteration, a robust tensor fit was calculated in which data frames with high residual error were excluded from the linear estimation of tensor model parameters. Outlier data frames (e.g., slices showing signal dropout due to sudden head movements) were replaced with values estimated from the tensor fit based on the censored data. More subtle forms of head motion were corrected using rigid body registration of each data frame with the corresponding eddy current-corrected volume (Hagler et al., 2009). The diffusion gradient matrix was adjusted for head rotation (Hagler et al., 2009; Leemans & Jones, 2009), and mean head motion values were calculated to correct for residual motion effects in group statistical analyses.

Spatial and intensity distortions caused by B_0_ field inhomogeneity were reduced using the reversing gradient method (Holland, Kuoperman, & Dale, 2010). Pairs of *b* = 0 (non-diffusion weighted) images with opposite phase encoding polarities were aligned using a nonlinear registration procedure, and the estimated displacement field volume was used to correct distortions in each successive diffusion-weighted volume. To use anatomical regions of interest (ROIs) from FreeSurfer's automated subcortical segmentation and cortical parcellation, *b* = 0 images were registered to T1-weighted structural images using mutual information (Wells et al., 1996) after coarse prealignment via within-modality registration to atlas brains. Diffusion images were resampled into a standard orientation with 1.7-mm isotropic voxel resolution. Standard-space registration was combined with the motion-correction registration, so that a single resampling step could be performed using cubic interpolation.

Several standard measures related to microstructural tissue properties were calculated after fitting the diffusion tensor, including fractional anisotropy, and mean, longitudinal (or axial), and transverse diffusivity. B values greater than 1,000 were excluded from tensor fitting to avoid need for nonlinear estimation, and diffusion tensor parameters were calculated using a linear estimation approach with log-transformed diffusion-weighted signals (Basser, Mattiello, & LeBihan, 1994; Le Bihan et al., 2001; Pierpaoli, Jezzard, Basser, Barnett, & Di Chiro, 1996).

Mean DTI measures were calculated for ROIs derived from FreeSurfer's automated segmentation and parcelation. To minimize partial volume effects, mean DTI measures for cortical ROIs were weighted based on the proportion of white versus gray matter within each voxel in an ROI, using information from the cortical surfaces generated by FreeSurfer during processing of T1-weighted images. A similar method was used to calculate weighted mean diffusivity values for subcortical ROIs, to minimize signal contamination due to cerebrospinal fluid partial voluming within voxels (Elman et al., 2017).

Resting-state fMRI data were head motion corrected by registering each frame to the first using AFNI's 3dvolreg (Cox, 1996). B0 distortions were corrected using the reversing gradient method (Holland et al., 2010). Displacement field was estimated from spin-echo field map scans, then adjusted for estimated between-scan head motion, and applied to gradient-echo images. Data were corrected for gradient nonlinearity distortions (Jovicich et al., 2006). Finally, between-scan motion correction was performed across all fMRI scans in imaging sessions. Registration was performed between T2-weighted, spin-echo B0 calibration scans and T1-weighted structural images using mutual information (Wells et al., 1996).

Following initial preprocessing, initial volumes were removed from the scan (Siemens/Philips: 8 TRs, GE DV25: 5 TRs, GE DV26: 16 TRs). Data were normalized and demeaned. Then, linear regression was performed to remove quadratic trends and signals correlated with motion and mean time courses of cerebral white matter, ventricles, and whole brain, plus first derivatives (Power et al., 2014; Satterthwaite et al., 2012). Motion regression included six parameters plus their derivatives and squares. Frames with displacement >0.3 mm were excluded from the regression (Power et al., 2014). Data were band-pass filtered between 0.009 and 0.08 Hz (Hallquist, Hwang, & Luna, 2013).

Preprocessed time courses were sampled onto the cortical surface projecting 1 mm into cortical gray matter along surface normal vector. Motion censoring was performed to reduce residual effects of head motion (Power et al., 2014; Power, Barnes, Snyder, Schlaggar, & Petersen, 2012). Motion estimates were filtered to attenuate signals (0.31–0.43 Hz) associated with respiration (18.6–25.7 respirations / minute). Time points with framewise displacement (FD) > 0.2 mm were excluded from variance and correlation calculations as well as time periods with <5 contiguous, subthreshold time points and time points that were outliers in *SD* across ROIs. Subcortical structures were labeled with atlas-based FreeSurfer segmentation (Fischl et al., 2002). Networks were defined as predefined groups of parcels stemming from functionally defined parcelation based on resting-state functional connectivity patterns (Gordon et al., 2016). A seed-based, correlational approach (Van Dijk et al., 2010) adapted for cortical surface based analysis (Seibert & Brewer, 2011) was performed. The average correlation between networks and ROIs is calculated as the average of the Fisher-transformed correlations.

The present study made use of tabulated data sheets resulting from these analyses; these were part of the first wave of released ABCD data. For all ACC modalities, because we had no hypotheses related to laterality effects, left and right, rostral and caudal ACC measures were averaged to create a summary ACC score. To create an average amygdala volume score, right and left amygdala volumes were averaged.

#### Quality assurance

FreeSurfer-processed structural data were available on 3,048 participants. We excluded 553 participants whose data sets were rated by the ABCD consortium as moderately or severely impacted by motion, intensity inhomogeneity, white matter underestimation, pial overestimation, or magnetic susceptibility artifact. Structural MRI analyses were performed on 2,495 participants.

Processed DTI data were available on 2,787 participants. We excluded 759 participants with more than 1.50 mm average framewise displacement, resulting in a total sample of 2,028 participants.

For the resting-state analyses, fully processed data were available on 2,727 participants. We further excluded 271 participants with a mean FD of >0.55 mm as well as 50 participants who had less than 4 min of resting-state data with FD < 0.20 mm and no fieldmap data collected within two scans prior to the resting-state scan, resulting in a sample of 2,164 for the resting-state fMRI analyses (Parkes, Fulcher, Yücel, & Fornito, 2018).

### Statistical analysis

Partial correlations were used to assess associations between the latent variables and child behavior scores correcting for child and parent sex, and child age. These statistics are meant as descriptors of the data, rather than tests of hypotheses. Therefore, no indices of inferential significance are reported. To test the hypothesized indirect effect of family environment on structure and function of the amygdala–mPFC circuit via pubertal stage, linear mediation analyses were performed in Mplus. Mediation analyses were corrected for child sex, age, and race. Brain measures were residualized for study site (dummy coded). Gray matter measures were additionally residualized for total brain volume (sum of left and right cortical volume and white matter volume). Due to the large scale of the cortical area and subcortical volume measures, these measures were converted to *z* scores. Separate models were run for each modality of the same structure (T1 ACC, T1 amygdala, DTI, and resting-state fMRI). Models were initially run on the full sample. To assess sex differences, we performed exploratory mediation analyses separately for boys and girls.

Because gray matter development follows an inverted U-shaped developmental trajectory with peaks of gray matter in adolescence (e.g., Giedd et al., 1999), we visualized the distribution of the gray matter metrics across pubertal stages (see Supplemental Figure S.2). Because the distribution of ACC cortical area and amygdala volume suggests that there could be a quadratic relation with pubertal stage, for these outcomes, quadratic mediation was also tested (quadratic association between mediator pubertal stage and gray matter outcome) using the Medcurve macro in SPSS (Hayes & Preacher, 2010). Whereas linear mediation analyses in Mplus can analyze categorical mediators, quadratic mediation in Medcurve can only handle continuous mediators. Therefore, for the quadratic mediation analyses, pubertal stage was regarded as a continuous variable and the original coding (Stage 1 through 4) was used. Contrary to linear mediation, where the indirect effect is similar for any level of the independent variable, in quadratic meditation, the effect size of the indirect effect depends on the level of the independent variable (Hayes & Preacher, 2010). Instantaneous indirect effects were assessed for the mean score and +/–1 *SD*. Bias-corrected bootstrapped confidence intervals (10,000 samples) were calculated to allow statistical inference.

## Results

Tables 1 and 2 provide sample characteristics for the sample used to create the family environment score (i.e., including individuals with poor-quality MRI data). As expected, there were significant differences in pubertal development between boys and girls, with more girls in higher stages compared to boys, χ^2^ (2) = 721.42, *p* < .001. Boys (*M* = –0.101, *SD* = 0.58) had a lower family environment score (e.g., lower quality environment) compared to girls (*M* = 0.00, *SD* = 0.56), *t* (3181) = 4.95, *p* < .001. Table 3 shows the correlations between the different latent variables (LVs) as well as the correlations between the LVs and the child behavioral measures. As can be seen, the overarching latent measure of family environment correlates similarly with all subordinate LVs (*r* = .76, *r* = .80, and *r* = .67 for the child-reported, parent-reported, and demographic LV, respectively). Contrary to the parent- and child-reported LVs, which show stronger correlations with the same reporter behavioral outcomes, the family environment LV shows a stable pattern of correlations with both parent- and child-reported child behavior measures.

**Table 1.** Sample characteristics; continuous variables

	*N*	*M*	*SD*	Range
Age (months)	3,183	119.42	7.25	107–132
Parental acceptance	3,178	2.79	0.29	1–3
Parental monitoring	3,182	4.41	0.50	2–5
Family conflict child	3,181	1.94	1.89	0–9
Family conflict parent	3,171	2.46	1.84	0–9
Parental psychopathology	3,114	37.58	17.19	0–142
Income	2,907	7.37	2.28	1–10
Family environment LV	3,183	–0.055	0.57	−2.19–1.41
Child-reported LV	3,183	–0.09	0.38	−3.23–1.67
Parent-reported LV	3,183	0.06	0.92	−1.50–2.88
Demographics LV	3,183	–0.03	0.94	−3.25–1.81
CBCL internalizing	3,183	5.22	5.44	0–49
CBCL externalizing	3,183	4.25	5.45	0–47
SDQ prosocial self	3,179	1.69	0.36	0–2
SDQ prosocial parent	3,178	1.75	0.40	0–2

*Note*: LV, latent variable. CBCL, Child Behavior Checklist. SDQ, Strengths and Difficulties Questionnaire.

**Table 2.** Sample characteristics; categorical variables

Categorical variable		*N*	*N* (%)
Boys		3,183	1,725 (54.19)
Race	Caucasian	1,797	1,797 (56.46)
	Black		285 (8.95)
	Hispanic		724 (22.74)
	Other		377 (11.85)
Pubertal stage	1	3,168	1,697 (53.31)
	2		765 (24.03)
	3+		706 (22.18)
Parental education	High school or less	3,138	495 (15.55)
	Some college		515 (16.18)
	Associate degree		393 (12.35)
	Bachelor's degree		945 (29.69)
	Master's degree		625 (19.64)
	Professional school/doctorate degree		210 (6.60)
Parental divorce/separation		3,151	981 (30.82)
Planned pregnancy		3,158	2,010 (63.15)

**Table 3.** Partial correlations between latent variables (LVs) and child behavior measures

	Child LV	Parent LV	Demographics	CBCL Int	CBCL Ext	SDQ prosocial self	SDQ prosocial parent
FE LV	.76	.80	.67	–.23	–.38	.21	.18
Child LV		.38	.31	–.13	–.25	.37	.17
Parent LV			.29	–.22	–.37	.08	.18
Demographics				–.15	–.22	.02	.00

*Note*: Correlations were corrected for child age and sex, and parent sex. FE, family environment latent variable. Child LV, latent variable representing child-reported data on parenting and family relationships. Parent LV, latent variable representing parent-reported data on family relationships. Demographics, latent variable representing family demographical information. CBCL, Child Behavior Checklist. Int, internalizing behavior. Ext, externalizing behavior. SDQ, Strengths and Difficulties Questionnaire.

Correlations between the outcomes of interest can be found in Table 4. All measures were residualized for scanning site (dummy coded) prior to calculating correlations. Gray matter measures were additionally residualized for total brain volume. See Supplemental Table S.1 for correlations between the motor-processing brain measures. For amygdala volume, ACC cortical thickness and cortical area, sex-corrected age correlations were *r* = .028, *p* = .167, *r* = –.124, *p* < .001, and *r* = .036, *p* = .072, respectively. Correlation between cingulo-opercular network–left and right amygdala functional connectivity and age (corrected for sex) were partial *r* = .007, *p* = .724, and partial *r* = .019, *p* = .349, respectively. Finally, for ACC fractional anisotropy, the sex-corrected correlation with age was partial *r* = .041, *p* = .062.

**Table 4.** Correlation between MRI measures

	ACC CA	ACC FA	Amygdala SV	CON-L amygdala FC	CON-R amygdala FC
ACC CT	–.082	–.072	.067	–.005	.028
ACC CA		–.257	–.017	.023	.002
ACC FA			–.049	.003	–.038
Amygdala SV				–.052	–.030
CON-L amygdala FC					.582

*Note*: All measures are residualized for data collection site. Gray matter measures were further residualized for total brain volume. ACC, anterior cingulate cortex. CT, cortical thickness. CA, cortical area. FA, fractional anisotropy. SV, subcortical volume. CON, cingulo-opercular network. L, left. R, right. FC, functional connectivity.

### Mediation analyses

**Figure 1.**
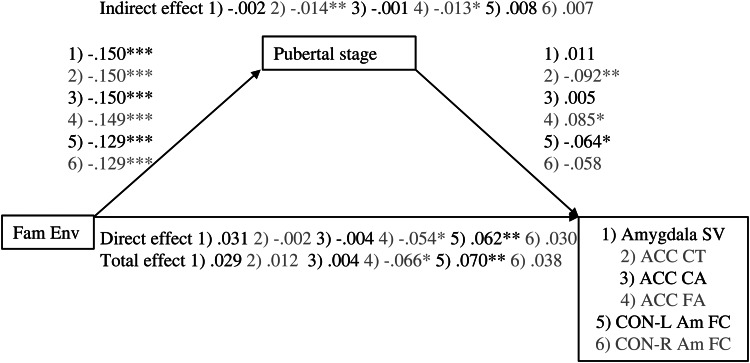
Associations between family environment and amygdala–mPFC measures are mediated by pubertal stage. Values are standardized coefficients. *p < .01. **p < .01. ***p < .001. Fam Env, family environment. ACC, anterior cingulate cortex. CT, cortical thickness. CA, cortical area. SV, subcortical volume. FA, fractional anisotropy. CON-Am FC, cingulu-opercular network–amygdala functional connectivity.

The results of the linear mediation analyses are presented in Figures 1 (total sample) and 2 (sex-stratified analyses). The full model details for the analyses of the amygdala–mPFC circuit can be found in Tables 5–7. In these tables, the top half describes the effect of predictors (family environment and pubertal stage) and confounding variables on the brain metric of interest. This part of the table thus provides information on the direct effect of family environment on brain structure or functioning as well as on the second leg of the indirect effect (pubertal stage on brain structure or function). The second half of these tables describes the effect of family environment and confounding variables on pubertal stage. As such, this part of the table provides information on the first leg of the indirect effect. For full model details of the quadratic mediation analyses, please see Supplemental Table S.2. For a description of the results of the motor circuit processing analyses, please see Supplemental Text 1 and Supplemental Tables S.3–S.6. For full-model estimates of the sex-stratified analyses, please see Supplemental Tables S.7–S.21.

**Figure 2.**
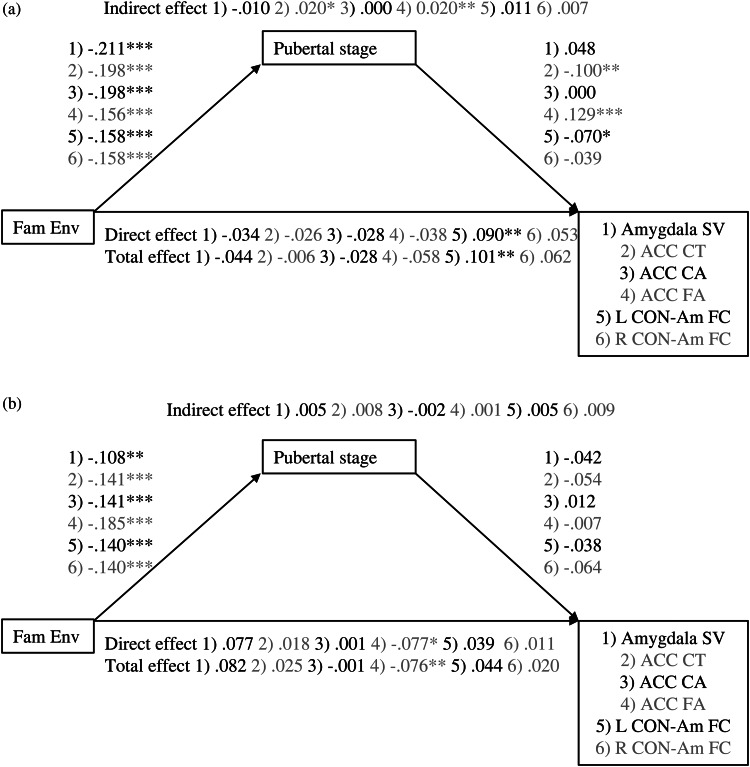
Mediation of association between family environment and amygdala–mPFC measures by pubertal stage for (a) girls and (b) boys. Values are standardized coefficients. *p < .01. **p < .01. ***p < .001. Fam Env, family environment. ACC, anterior cingulate cortex. CT, cortical thickness. CA, cortical area. SV, subcortical volume. FA, fractional anisotropy. CON-Am FC, cingulu-opercular network–amygdala functional connectivity.

**Table 5.** Mediation model parameters: Anterior cingulate cortical thickness and area

	ACC cortical thickness				ACC cortical area				Amygdala volume			
	β	*SE*	β/*SE*	*p*	β	*SE*	β /*SE*	*p*	β	*SE*	β /*SE*	*p*
Fam Env^a^	–0.002	0.020	–0.105	.916	–0.004	0.022	–0.160	.873	0.054	0.036	1.495	.135
Pubertal^b^ stage	–0.092	0.032	–2.899	.004	0.005	0.031	0.147	.883	0.009	0.024	0.379	.705
Age	–0.105	0.021	–4.982	<.001	0.035	0.021	1.646	.100	0.003	0.003	1.060	.289
Sex	–0.111	0.026	–4.243	<.001	–0.090	0.025	–3.603	<.001	0.261	0.050	5.217	<.001
Race	–0.020	0.020	–0.980	.327	0.000	0.020	0.001	.999	0.012	0.019	0.627	.530
	Outcome: pubertal stage				Outcome: pubertal stage				Outcome: pubertal stage			
Fam Env^c^	–0.150	0.020	–7.442	<.001	–0.150	0.020	–7.442	<.001	–0.322	0.044	–7.358	<.001
Age	0.218	0.019	11.392	<.001	0.218	0.019	11.392	<.001	0.037	0.004	10.407	<.001
Sex	–0.511	0.015	–33.433	<.001	–0.511	0.015	–33.434	<.001	–1.256	0.050	–24.953	<.001
Race	0.136	0.020	6.807	<.001	0.136	0.020	6.807	<.001	0.149	0.022	6.925	<.001

*Note*: ACC, anterior cingulate cortex. Fam Env, family environment. ^a^Direct effect. ^b^Indirect effect Step 2. ^c^Indirect effect Step 1.

**Table 6.** Mediation model parameters: Anterior cingulate white matter fractional anisotropy

	*Β*	*SE*	β/*SE*	*p*
Family environment^a^	–0.054	0.023	–2.329	.020
Pubertal stage^b^	0.085	0.033	2.569	.010
Age	0.027	0.023	1.166	.244
Sex	–0.018	0.027	–0.670	.503
Race	0.036	0.023	1.565	.118
Outcome: pubertal stage				
Family environment^c^	–0.149	0.022	–6.894	<.001
Age	0.213	0.022	9.609	<.001
Sex	–0.507	0.017	–29.495	<.001
Race	0.141	0.021	6.695	<.001

^a^Direct effect. ^b^Indirect effect Step 2. ^c^Indirect effect Step 1.

**Table 7.** Mediation model parameters: Cinculo-opercular network–amygdala connectivity

	CON–Left amygdala				CON–Right amygdala			
	β	*SE*	β/*SE*	*p*	β	*SE*	β /*SE*	*p*
Family environment^a^	0.062	0.022	2.795	.005	0.030	0.022	1.337	.181
Pubertal stage^b^	–0.064	0.032	–2.030	.042	–0.058	0.031	–1.834	.067
Age	0.016	0.023	0.722	.470	0.029	0.022	–1.360	.174
Sex	–0.041	0.027	–1.534	.125	–0.062	0.027	–2.316	.021
Race	–0.041	0.020	–2.040	.041	–0.037	0.020	1.856	.063
	Outcome: pubertal stage				Outcome: pubertal stage			
Family environment^c^	–0.129	0.020	–6.530	<.001	–0.129	0.020	–6.530	<.001
Age	0.140	0.020	11.483	<.001	0.140	0.020	11.483	<.001
Sex	–0.508	0.015	32.913	<.001	–0.508	0.015	32.913	<.001
Race	0.140	0.020	7.131	<.001	0.140	0.020	7.131	<.001

*Note*: CON, cingulo-opercular network. ^a^Direct effect. ^b^Indirect effect Step 2. ^c^Indirect effect Step 1.

### Gray matter structure

#### Amygdala volume

For amygdalae volume, the total, direct (effect controlled for pubertal stage), and indirect effects (effect via pubertal stage) of family environment were not significant, β = 0.029, *p* = .147, β = 0.031, *p* = .135, β = –0.002, *p* = .707, nor was there evidence for quadratic mediation. The quadratic unstandardized association between pubertal stage and amygdala volume was not significant (*b* = –0.039, *p* = .268).

#### Cortical thickness

The indirect effect of family environment on ACC cortical thickness through pubertal stage was significant, β = 0.014, *p* = .007, but the total and direct effects were not, β = 0.012, *p* = .552, β = –0.002, *p* = .916, respectively. Thus, a lower quality family environment was related to a thinner ACC, but only through its association with a more advanced pubertal stage.

#### Cortical area

No significant linear associations were found for ACC cortical area (β = –0.004, *p* = .843, β = –0.004, *p* = .872, β = –0.001, *p* = .885, for the total, direct, and indirect effects, respectively). However, the quadratic mediation model for ACC cortical area did provide some evidence for mediation. There was a negative quadratic association between pubertal stage and ACC cortical area (*b* = –0.098, *p* = .006), corresponding with the inverted U-shaped developmental trajectories previously reported. However, none of the instantaneous indirect effects were significant, *b* = –0.005, 95% CI [–0.015, 0.004], *b* = –0.007, 95% CI [0.019, 0.002], *b* = –0.010, 95% CI [–0.023, 0.001], for –1 *SD*, mean, and +1 *SD*, respectively. As the indirect effect seems to increase with increasing family environment score, these results suggest that for children from very low-stress family environments only, ACC cortical area is decreased through its association with a less advanced pubertal stage.

#### Sex-specific effects

The exploratory analyses stratified by sex suggest that the significant ACC cortical thickness association was driven mainly by the girls in the sample. In girls, the indirect effect of family environment through pubertal stage was significant, β = 0.0.20, *p* = .013, but total and direct effects were not, β = –0.006, *p* = .840, β = –0.026, *p* = .402. For boys, the total, direct, and indirect effects were not significant (β = 0.006, *p* = .366, β = 0.004, *p* = .533, β = 0.002, *p* = .181, respectively).

Similar to the analyses in the total sample, the stratified linear mediation analyses of ACC cortical area did not provide any significant results (ACC cortical area: girls: β = –0.028, *p* = .354, β = –0.028, *p* = .358, β = 0.000, *p* = .998, for total, direct, and indirect effects, respectively; boys β = –0.001, *p* = .987, β = 0.002, *p* = .970, β = –0.003, *p* = .755, for total, direct, and indirect effects, respectively). In girls, the quadratic association between pubertal stage and ACC cortical area was significant, *b* = –0.131, *p* = .004. However, again no significant instantaneous indirect effects were found, *b* = 0.013, 95% CI [–0.002, 0.034], *b* = 0.006, 95% CI [–0.009, 0.024], *b* = –0.005, 95% CI [–0.019, 0.015], for –1 *SD*, *mean*, and +1 *SD*, respectively. In boys, no evidence for quadratic mediation was found (quadratic association between pubertal stage and ACC cortical area: *b* = –0.027, *p* = .676).

The stratified linear analyses on amygdala volume were not significant (girls: β = –0.044, *p* = .393, β = –0.034, *p* = .546, β = –0.10, *p* = .586, for total, direct, and indirect effects, respectively; boys β = 0.082, *p* = .088, β = 0.077, *p* = .097, β = 0.005, *p* = .660, for total, direct, and indirect effects, respectively). The quadratic mediation model provided no evidence for quadratic mediation in girls (quadratic association between pubertal stage and amygdala volume: *b* = –0.053, *p* = .215) or boys (quadratic association between pubertal stage and amygdala volume: *b* = –0.048, *p* = .741).

### White matter integrity: Fractional anisotropy

In the total sample, the total, direct, and indirect effects through pubertal stage of family environment on ACC fractional anisotropy were significant β = –0.066, *p* = .003, β = –0.054, *p* = .020, β = –0.013, *p* = .015, respectively. Thus, a lower quality family environment related to higher ACC fractional anisotropy. This association was partly mediated by a higher pubertal stage.

The stratified analyses suggest that for girls, the family environment associates with ACC fractional anisotropy only through its association with pubertal stage (β = –0.058, *p* = .107, β = –0.038, *p* = .298, β = –0.020, *p* = .009, for total, direct, and indirect effects, respectively), whereas for boys, only the direct effect of family environment on ACC fractional anisotropy was significant (β = –0.076, *p* = .005, β = –0.077, *p* = .007, β = 0.001, *p* = .868, for total, direct, and indirect effects, respectively).

### Resting-state fMRI

In the total sample, the total, direct, and indirect effects of family environment on cingulo-opercular network–left amygdala functional connectivity were β = 0.070, *p* = .001, β = 0.062, *p* = .005, β = 0.008, *p* = .055, respectively. For cingulo-opercular network–right amygdala functional connectivity, the total, direct, and indirect effects were β = 0.038, *p* = .086, β = 0.030, *p* = .181, β = 0.007, *p* = .080, respectively. Thus, family environment was positively associated with cingulo-opercular network–amygdala functional connectivity. For both left and right amygdala– cingulo-opercular network functional connectivity, the indirect effect of family environment on functional connectivity via pubertal stage indicated a trend in the expected direction.

The exploratory analyses stratified by sex suggest that the total and direct effects of family environment on cingulo-opercular network–left amygdala functional connectivity were significant for girls, whereas a trend was found for the indirect effect (β = 0.101, *p* = .001, β = 0.090, *p* = .004, β = 0.011, *p* = .064, respectively). For boys, no significant effects were found (β = 0.044, *p* = .133, β = 0.039, *p* = .198, β = 0.005, *p* = .37, respectively). For cingulo-opercular network–right amygdala functional connectivity, no significant effects were found for girls nor boys (girls: β = 0.059, *p* = .076, β = 0.121, *p* = .099, β = 0.006, *p* = .306, for total, direct, and indirect effects, respectively; boys β = 0.020, *p* = .518, β = 0.011, *p* = .738, β = 0.009, *p* = .151, for total, direct, and indirect effects, respectively).

### Motor-processing areas

No significant indirect effects were found for cortical structure, white matter integrity, or functional connectivity of the motor circuit. Only a significant direct and total effect of family environment on functional connectivity between the left amygdala and somatomotor–mouth network were found, β = 0.061, *p* = .003, β = 0.062, *p* = .003, respectively.

## Discussion

The present study examined whether associations between the quality of a child's family environment and measures of the amygdala–mPFC circuit's structure and function were mediated by pubertal development. For ACC cortical thickness, cortical area, and fractional anisotropy, evidence was found that a more stressful family environment relates to brain structure through accelerated pubertal development. For amygdala–cingulo-opercular network resting-state functional connectivity, results indicated a trend in the expected direction. There were no direct or indirect associations between family environment and amygdala volume. Original studies reporting accelerated development of the amygdala–mPFC circuit focused on functional MRI data only. Our findings provide suggestive evidence from a population-based sample that accelerated development of the amygdala–mPFC circuit in response to family-related stress may not be limited to brain activation (similar relations are found for mPFC [ACC] gray and white matter structure) and may be (in part) the consequence of accelerated pubertal development.

Cortical gray matter follows an inverted U-shaped developmental trajectory: during childhood, gray matter increases until it peaks in adolescence and declines thereafter (e.g., Giedd et al., 1999). White matter has been shown to increase over age until at least the late 20s (Westlye et al., 2010). Our results suggest that a suboptimal family environment relates to higher ACC white matter integrity, which can be seen as a more mature state of brain development given the age range of the current sample. This association was mediated by a more advanced pubertal stage. Similarly, through a more advanced pubertal stage, a suboptimal family environment related to a thinner cortex, which—as gray matter decreases over pubertal development—can be seen as a more mature state. For ACC cortical area, the indirect effect is more complicated due to its quadratic nature, but coheres with the hypothesized model. As such, a suboptimal family environment may accelerate ACC gray and white matter development.

Although a large literature exists on the association between a child´s familial context and pubertal development, to our knowledge, no studies have examined the consequences of these findings for individual differences in patterns of brain structure and function. Similarly, accelerated development of the amygdala–mPFC circuit has not been ascribed to accelerated pubertal development, but is regarded as a direct effect of the environment or stress on this circuit (Callaghan & Tottenham, 2016; Gee, Gabard-Durnam, et al., 2013). In rats, early life stress (through increasing cortisol) has been associated with precocious engagement of the amygdala in response to odor-shock conditioning (Moriceau & Sullivan, 2006). In their study on accelerated development of the amygdala–mPFC circuit in previously institutionalized children, Gee, Gabard-Durnam, et al. (2013) show that the association between institutionalized care and accelerated development of amygdala–mPFC circuit is mediated by cortisol levels. The authors suggest that the accelerated development of the amygdala–mPFC circuit may be a result of early life increased cortisol levels resulting in earlier amygdala hyperactivity, which in its turn may facilitate development of connections with the mPFC. Further, in rodents and primates, the amygdala has been suggested to play a role in regulating the onset of puberty (Adekunbi et al., 2017; Li et al., 2015; Stephens, Raper, Bachevalier, & Wallen, 2015). This particular role of the amygdala bridges the theory that the precocious development of the amygdala–mPFC circuit is a consequence of stress (cortisol) affecting the amygdala and the theory presented here, that accelerated development of the amygdala–mPFC circuit is (in part) a consequence of accelerated pubertal development.

In a previously institutionalized sample, accelerated neural development was reported in children aged 6.5–10.4 years (Gee, Gabard-Durnam, et al., 2013). Given the age of the sample, one might argue that pubertal development is an unlikely driving force of such development. However, adrenarche, which is considered the first stage of pubertal development, typically occurs around 6–8 years of age (Campbell, 2006; McClintock & Herdt, 1996), and if accelerated, may well precede the accelerated neural development reported by Gee, Gabard-Durnam, et al., 2013. Adrenal hormones are continuously involved in pubertal development and affect, for example, pubic hair development and pubertal skin changes, pubertal indices measured in the Pubertal Development Scale utilized here to study pubertal stage. Adrenarche affects brain development (for a review, see Byrne et al., 2017), and although considered distinct from gonadarche, through its influence on GABA neurons adrenarche may indirectly affect gonadarche (Genazzani, Bernardi, Monteleone, Luisi & Luisi, 2000). Like gonadarche and menarche, adrenarche has been reported to be accelerated in response to early life stress (e.g., Belsky et al., 2015; Ellis & Essex, 2007), and children experiencing early adrenarche are more likely to experience early pubarche (adrenal), menarche, and telarche (gonadal; Ibáñez, Jiménez, & de Zegher, 2006; Liimatta, Utriainen, Voutilainen, & Jääskeläinen, 2017; Pereira, Iñiguez, Corvalan, & Mericq, 2017). The synthesis and release of both cortisol and dehydroepiandrosterone (DHEA), a hormonal index of adrenarcheal development, are triggered by the same hormone, ACTH. Moreover, DHEA plays a role in the stress system (Saczawa, Graber, Brooks-Gunn, & Warren, 2013), providing a direct biological link between stress and adrenal hormones. As such, adrenarche is an early marker of pubertal onset that predates the release of gonadal hormones. We speculate that it may play a role in the accelerated neural development that has been observed across studies in response to early stress. Of course, the family environment could affect brain development via multiple pathways: cortisol could directly affect the amygdala–mPFC circuit as previously suggested, and simultaneously have a similar effect through its association with pubertal development and hormones (Ellis et al., 1999; Graberc et al., 1995; Romans et al., 2003). This hypothesized mechanism of action merits further scrutiny given that there is inconsistent evidence for accelerated pubertal development in previously institutionalized girls.

When the quality of parental care and the familial environment is low, it may (from an evolutionary standpoint) be adaptive to reach a reproductive age sooner. Similarly, when parental care is limited, it may be beneficial to develop some form of self-regulation skills sooner instead of relying on parents for emotion regulation. In addition, the accelerated development of emotion regulation skills can be regarded in light of evolutionary adaptiveness: an individual capable of regulating his or her emotions may be better equipped to find a partner. Nevertheless, although accelerated development may be beneficial in the short run, a long childhood period has reported benefits for development. For example, superior intelligence has been associated with prolonged cortical thickness growth in children (Shaw et al., 2006). The earlier termination of childhood may result in suboptimal development (e.g., a higher risk for health and adjustment problems or psychopathology later in life; Kelsey, Gammon, & John, 1993; Winer, Powers, & Pietromonaco, 2017).

Contrary to psychosocial acceleration theory, child development theory (Ellis, 2004) suggests that information from the environment is used to coordinate the length of the childhood period without focusing on reproduction. This theory poses that, if the environment is positive, the child can take its time to reap the benefits of a long childhood. When the family environment is suboptimal, earlier independence from the parents may be preferred and the child accelerates his or her pubertal development. This, however, does not mean that the child will also engage in sexual behavior and reproduction at an earlier age. Our results for ACC cortical area are more in line with this latter theory, as they suggest that pubertal and brain development is delayed in children from low-stress families, rather than accelerated in high-stress families. However, despite including the full range of pubertal development, our sample includes relatively few children in pubertal Stages 4 and 5. In order to draw strong conclusions regarding quadratic mediation, a more equal distribution of pubertal stage should be evident. Nevertheless, our results suggest the possibility of quadratic mediation, which may provide a worthwhile avenue to explore as this sample develops further.

The associations between family environment or pubertal stage and amygdala–cingulo-opercular network functional connectivity were not significant but indicated a trend in the expected direction. Although our hypotheses were derived from work examining task-based functional connectivity, here, the functional connectivity data provided the least compelling results. Because ABCD did not release amygdala–mPFC functional connectivity metrics, we selected amygdala–cingulo-opercular functional connectivity as the best available proxy for amygdala–mPFC functional connectivity. However, the cingulo-opercular network only includes part of the mPFC as well as regions outside of the mPFC, and these characteristics may be critical in explaining why our results are less strong than previously reported findings (Gee, Gabard-Durnam, et al., 2013; Thijssen et al., 2017). In addition, no associations between family environment or pubertal stage and amygdala volume were found. Although some studies report continued amygdala structural development through childhood (Goddings & Giedd, 2014; Neufang et al., 2009), other studies suggest that the amygdala matures prior to adolescence (Giedd et al., 1996), which would explain why no associations with pubertal stage were found in the present study. Moreover, although evidence for changes in amygdala volume in relation to early adversity has been consistently reported (Mehta et al., 2009; Tottenham et al., 2010), studies on normal variation in family environment and amygdala volume have provided mixed results (Kok et al., 2015; Whittle et al., 2014). Alternatively, T1 MRI measures cortical thickness and surface area much more precisely than the volumes of subcortical structures. Thus, the null effect for amygdala volume might simply reflect lesser precision of MRI measurement, as compared to cortical surface measures. The mediation models of motor processing largely provided nonsignificant results, except for a direct effect of the child's family environment on left amygdala–somatomotor-mouth network functional connectivity. As the motor cortex is one of the first brain regions to reach peak cortical thickness/area (Giedd et al., 1999), we did not expect mediation by pubertal stage in these regions, providing some evidence for the specificity of our results.

Similar to previous literature, our results suggest that girls initiate puberty earlier than boys (Petersen & Crockett, 1985), and that Caucasian children are later to initiate puberty than children from other races (Butts & Seifer, 2010). Our exploratory analyses stratified by sex suggest that direct and indirect associations between family environment and the amygdala–mPFC circuit were more prominent for girls than for boys. These findings are in line with studies supporting psychosocial acceleration theory, which focuses on girls rather than boys. Similarly, the exploratory analyses of Thijssen et al. (2017) suggest that acceleration of functional development of the amygdala–mPFC circuit may be present only in girls. Because females are physically more restricted in the number of offspring they can conceive relative to males, it makes evolutionary sense that girls specifically mature sooner. Moreover, rodent studies suggest that the reorganizing effects of puberty are smaller in males than in females, which may explain our sex-stratified results (Juraska & Willing, 2017). Alternatively, as pubertal development is slower in boys than in girls, it is possible that associations between pubertal stage and brain development in boys simply are not yet captured in our sample but will appear at a later age. Nevertheless, in girls, precocious puberty has been associated with increased depression and anxiety later in adolescence (Mendle, Turkheimer, & Emery, 2007; Wichstrom, 2000). As the amygdala–mPFC circuit has been implicated in emotional problems (Holmes et al., 2012), the co-occurrence of accelerated pubertal and amygdala–mPFC circuit development in girls specifically may be able to account for these findings.

Research in the medical field suggests that stressful environments may accelerate aging through telomere length. Telomeres are the protective caps at the end of chromosomes that erode with every cell division until a state of senescence has been reached (López-Otín, Blasco, Partridge, Serrano, & Kroemer, 2013). Accelerated telomere erosion can thus be seen as accelerated aging. Initial studies focused on adults and elderly (Epel et al., 2004; Tomiyama et al., 2012), but even in children and infants, studies suggest effects of stress on telomere erosion (Drury et al., 2012; Entringer et al., 2013; Shalev et al., 2013). Similar to accelerated pubertal development, the pathway from early adversity to accelerated telomere erosion may include hypothalamic–pituitary–adrenal axis activation and epigenetics (Kroenke et al., 2011; Tomiyama et al., 2012), but also associations with inflammation, oxidative stress, and mitochondrial regulation have been reported (Cai et al., 2015; Epel et al., 2004; Jurk et al., 2014; Tyrka et al., 2016). From a medical perspective, results of accelerated telomere erosion have been interpreted as the consequence of wear and tear on biological systems caused by stress. Belsky and Shalev (2016) argue that this line of work on accelerated telomere erosion and the line of work on accelerated pubertal development may be related, and makes a case for interpreting telomere erosion from an evolutionary rather than a medical perspective (i.e., the organism adaptively trades-off longer term health and longevity for increased probability of reproducing before dying by maturing early). The results presented here add to this line of thinking and suggest that the family environment may relate to a cascade of biological events that result in accelerated aging more generally. Future research may take an interest in studying associations between family adversity, telomere erosion, and brain development as well as in examining the role of processes such as epigenetics and inflammation in the association between family environment and brain development.

Besides the hypothesized indirect effects, some potentially interesting direct effects of the child's family environment on brain function and structure were found. The sex-stratified analyses suggested increased ACC fractional anisotropy in children from lower quality family environments for both sexes. However, whereas for girls only an indirect association via pubertal stage was found, for boys only a direct effect was found, possibly suggesting accelerated development unrelated to pubertal development. Alternatively, this finding could indicate that boys from lower quality family environments have higher fractional anisotropy values independent of developmental rate. In addition, a more positive family environment was directly related to increased somatomotor-mouth network–left amygdala functional connectivity. As we did not find significant associations between functional connectivity of the somatomotor-mouth network and the left amygdala and age or pubertal stage, this finding cannot be interpreted in light of accelerated or possible slowed development in response to adversity, but suggests that a child's family environment can have broad and diverse effects on child brain development while pubertal effects may be more specific.

Several limitations of the present study should be noted. Although the ABCD Study is a longitudinal cohort study, at present only data from the first data wave, on half of the final baseline sample of 11,875 children, are available. As such, the present study is a cross-sectional study, and any statements regarding developmental trajectories should be considered speculative. Similarly, as predictor, outcome, and mediator variables are reported at the same time, the data set in its current form is not ideal for examining mediation. In addition, the original study reporting accelerated neural development in previously institutionalized children reported a developmentally mature phenotype in adolescents aged 10.5–17.6 years (Gee, Gabard-Durnam, et al., 2013). If amygdala–mPFC development is considered complete by the time the adultlike fear processing phenotype (negative connectivity vs. positive connectivity) is evident, then the current study's sample of 9- to 10-year-old children would not be optimal to study accelerated development. However, the negative connectivity pattern observed in early adolescents may differ still from the negative pattern observed later in development (e.g., in adults). For example, Wu et al. (2016) report decreased amygdala–mPFC functional connectivity in response to emotional faces in adults versus adolescents. Unlike the shift from positive to negative functional connectivity reported in relation to fear processing, mPFC structural development is more linear and protracted. As such, the age of the ABCD sample does not pose a direct problem for our conceptual model. Although family environment has been shown to be relatively stable in children over time (Loeber et al., 2000), our measure of family environment does not necessarily reflect early family functioning. This is particularly important, because previous studies on accelerated pubertal and amygdala–mPFC development report associations for early family environment (e.g., Gee, Gabard-Durnam, et al., 2013; Romans et al., 2003; Thijssen et al., 2017). Moreover, behavioral changes associated with puberty have been shown to affect family relationships. Thus, we cannot be certain about the causal relationship of the association between family environment and pubertal development, as there is evidence supporting a bidirectional pattern of influence (family environment accelerating pubertal development *and* pubertal development affecting family relationships). However, because our family environment variable includes measures other than parent–child conflict such as socioeconomic status, the inverse relationship (i.e., pubertal development affecting the family environment score) may be less salient. Thus, although it does not capture all possible features of family function, our measure of family environment presents an important predictor of pubertal and neural development. Moreover, as it is our hypothesis that family-related stress accelerates development, our family environment variable combines different factors including family dynamics and socioeconomic factors. However, this could also be considered a limitation as our approach did not test for differential effects of different types of variables. In addition, due to the population-based nature of the sample, levels of adversity are relatively low. Relatedly, due to the large sample size of the present study, small effects (such as the results presented here) may become significant. However, at the population level, even small effect sizes are potentially meaningful. Given that the pattern of results coheres across MRI modalities, confidence in the significance of our findings is increased.

In conclusion, despite the limited range in pubertal development within this sample of 9- to 10-year-olds, our results provide suggestive cross-sectional evidence that the association between family environment and accelerated development of the amygdala–mPFC circuit's structure and structural connectivity may be in part explained by accelerated pubertal development. These associations appear stronger in girls than in boys and may play a role in mental health problems observed after precocious puberty. If accelerated development of the amygdala–mPFC circuit is the consequence of an earlier onset of puberty, other brain regions and circuits that are involved in affective regulation would be expected to portray similar trajectories of accelerated development and may be related to adolescent mental health. In a recent small study, early life stress was associated with increased gray matter reductions in a variety of cortical and subcortical brain regions from ages 14 to 17 (Tyborowska et al., 2018). Such associations should be further explored in future studies. Although currently only cross-sectional data are available, ABCD is a longitudinal study from age 9–10 to adulthood. Thus, once longitudinal data are available, our cross-sectional results should be replicated longitudinally. Moreover, hormonal measures could be used in future analyses to support interpretations. Although the present study provides important insight on development in relation to family-related stress, such longitudinal studies are the necessary next steps in unraveling the effects of the family environment on child brain maturation.

## Supplementary material

For supplementary material accompanying this paper visit https://doi.org/10.1017/S0954579419000580. click here to view supplementary material
