# Pubertal development mediates the association between family environment and brain structure and function in childhood – ADDENDUM

**DOI:** 10.1017/S0954579420000322

**Published:** 2021-02

**Authors:** Sandra Thijssen, Paul F. Collins, Monica Luciana

In November 2019, the Adolescent Brain and Cognitive Development consortium communicated that previously released functional MRI data from Philips scanners has been processed incorrectly and should not be analyzed. The resting-state fMRI analyses reported in Thijssen et al. (2019) include data from Philips scanners. We have reanalyzed our resting-state fMRI data excluding participants scanned on a Philips scanner (n = 256). Excluding the Philips data did not significantly affect our results. For the new results, please see below. The conclusions described in the manuscript remain unchanged.

## Resting-state fMRI

In the total sample excluding those scanned with Phillips scanners, the total, direct, and indirect effects of Family Environment on cingulo-opercular network–left amygdala functional connectivity were β = 0.068, *p* = .003, β = 0.059, *p* = .010, β = 0.009, *p* = .071, respectively. For cingulo-opercular network–right amygdala functional connectivity, the total, direct, and indirect effects were β = 0.044, *p* = .055, β = 0.036, *p* = .122, β = 0.008, *p* = .106, respectively. Thus, Family Environment was positively associated with cingulo-opercular network–amygdala functional connectivity. For the left amygdala–cingulo-opercular network functional connectivity, the indirect effect of family environment on functional connectivity via pubertal stage indicated a trend in the expected direction. For right amygdala–cingulo-opercular network functional connectivity, the indirect effect no longer indicates a trend (*p >* .1). As the effect size of the indirect effect increased from β = 0.007 to β = 0.008 when excluding the Philips data, this difference is solely explained by decreased power.

The exploratory analyses stratified by sex suggest that the total and direct effects of Family Environment on cingulo-opercular network–left amygdala functional connectivity were significant for girls, whereas a trend was found for the indirect effect (β = 0.090, *p* = .005, β = 0.078, *p* = .017, β = 0.012, *p* = .093, respectively). For boys, no significant effects were found (β = 0.049, *p* = .112, β = 0.044, *p* = .157, β = 0.005, *p* = .459, respectively). For cingulo-opercular network–right amygdala functional connectivity, no significant effects were found for girls nor boys (girls: β = 0.061, *p* = .071, β = 0.053, *p* = .132, β = 0.008, *p* = .226 for total, direct, and indirect effects, respectively; boys β = 0.030, *p* = .322, β = 0.023, *p* = .459, β = 0.007, *p* = .289, for total, direct, and indirect effects, respectively).

**Table 6.** Mediation model parameters––Cinculo-opercular network–amygdala connectivity

	CON–left amygdala				CON–right amygdala			
	β	*S.E.*	β /*S.E*.	*p*	β	*S.E.*	β /*S.E.*	*p*
Family Environment^∞^	0.059	0.023	2.582	.010	0.036	0.023	1.548	.122
Pubertal stage^+^	−0.064	0.033	−1.923	.054	−0.055	0.033	−1.692	.091
Age	0.002	0.023	0.106	.915	0.021	0.024	0.878	.380
Sex	−0.031	0.027	−1.160	.246	−0.055	0.028	−1.984	.047
Race	−0.040	0.023	−1.768	.077	−0.032	0.021	−1.531	.126
	Outcome: Pubertal stage				Outcome: Pubertal stage			
Family Environment^*f*^	−0.136	0.022	−6.189	<.001	−0.136	0.022	−6.189	<.001
Age	0.231	0.021	11.159	<.001	0.231	0.021	11.159	<.001
Sex	−0.504	0.016	−30.950	<.001	−0.504	0.016	−30.950	<.001
Race	0.136	0.020	6.686	<.001	0.136	0.020	6.686	<.001

*Note:* CON = cingulo-opercular network; ^∞^ = direct effect; ^+^ = indirect effect Step 2; ^*f*^ = indirect effect Step 1.

**TableS9.** Mediation model parameters––Cinculo-opercular network–amygdala connectivity in girls

	CON–left amygdala				CON–right amygdala			
	β	*S.E.*	β /*S.E*.	*p*	β	*S.E.*	β /*S.E.*	*p*
Family Environment^∞^	0.078	0.033	2.391	.027	0.053	0.035	1.505	.132
Pubertal stage^+^	−0.071	0.039	−1.816	.069	−0.049	0.039	−1.261	.207
Age	−0.048	0.034	−1.404	.160	0.014	0.031	0.416	.677
Race	−0.044	0.036	−1.209	.227	−0.050	0.033	−1.637	.102
	Outcome: Pubertal stage				Outcome: Pubertal stage			
Family Environment^*f*^	−0.171	0.034	−5.058	<.001	−0.171	0.034	−5.058	<.001
Age	0.300	0.030	9.991	.001	0.300	0.030	9.991	.001
Race	0.111	0.034	3.295	<.001	0.111	0.034	3.295	<.001

*Note:* ^∞^ = direct effect; ^+^ = indirect effect Step 2; ^*f*^ = indirect effect Step 1; CON = cingulo-opercular network.

**TableS12.** Mediation model parameters––Cinculo-opercular network–amygdala connectivity in boys

	CON-–eft amygdala				CON–right amygdala			
	β	*S.E.*	β /*S.E*.	*p*	β	*S.E.*	β /*S.E.*	*p*
Family Environment^∞^	0.044	0.031	1.415	.157	0.023	0.031	0.740	.459
Pubertal stage^+^	−0.034	0.043	−0.794	.427	−0.048	0.042	−1.146	.252
Age	0.042	0.032	1.298	.194	0.027	0.030	0.900	.368
Race	−0.038	0.029	−1.286	.198	−0.016	0.029	−0.554	.579
	Outcome: Pubertal stage				Outcome: Pubertal stage			
Family Environment^*f*^	−0.142	0.036	−3.960	<.001	−0.142	0.036	−3.960	<.001
Age	0.223	0.035	6.289	<.001	0.223	0.035	6.289	<.001
Race	0.212	0.031	6.846	<.001	0.212	0.031	6.846	<.001

*Note:* ^∞^ = direct effect; ^+^ = indirect effect Step 2; ^*f*^ = indirect effect Step 1; CON = cingulo-opercular network.

## Somato-motor mouth network–amygdala functional connectivity

For the resting-state model with motor processing measures, onlythe total and direct effects of Family Environment on SOMM–left amygdala FC were significant (β = 0.060, *p* = .005, β = 0.061, *p* = .006, respectively), but not the indirect effect (β = −0.001, *p* = .847). No associations between Family Environment and SOMM–right amygdala were found (β = −0.013, *p* = .583, β = −0.015, *p* = .542, β = 0.002, *p* = .693, for total, direct, and indirect effects, respectively).

For the resting-state model with motor processing measures, in girls the total and direct effects, and in boys only the total effect of Family Environment on SOMM–left amygdala FC were significant (girls: β = 0.056, *p* = .048, β = 0.064, *p* = .036, β = −0.008, *p* = .302 for total, direct, and indirect effects, respectively; boys β = 0.063, *p* = .041, β = 0.056, *p* = .074, β = 0.006, *p* = .357. for total, direct, and indirect effects, respectively). No significant associations were found between Family Environment and SOMM–right amygdala (girls: β = 0.027, *p* = .421, β = 0.026, *p* = .452, β = 0.001, *p* = .886 for total, direct, and indirect effects,respectively; boys β = −0.043, *p* = .157, β = −0.046, *p* = .141, β = 0.003, *p* = .606 for total, direct, and indirect effects, respectively).

**TableS6.** Mediation model parameters––Somatomotor-mouth network–amygdala connectivity

	SOMM–left amygdala				SOMM–right amygdala			
	β	*S.E.*	β /*S.E*.	*p*	β	*S.E.*	β /*S.E.*	*p*
Family Environment^∞^	0.061	0.022	2.767	.006	−0.015	0.024	−0.610	.542
Pubertal stage^+^	0.007	0.035	0.199	.843	−0.013	0.033	−0.404	.686
Age	−0.021	0.023	−0.925	.355	−0.011	0.025	−0.437	.662
Sex	−0.009	0.029	−0.309	.757	0.007	0.026	0.279	.780
Race	−0.015	0.022	−0.678	.498	−0.026	0.022	−1.182	.237
	Outcome: Pubertal stage				Outcome: Pubertal stage			
Family Environment^*f*^	−0.136	0.022	−6.189	<.001	−0.136	0.022	−6.189	<.001
Age	0.231	0.021	11.159	<.001	0.231	0.021	11.159	<.001
Sex	−0.504	0.016	−30.950	<.001	−0.504	0.016	−30.950	<.001
Race	0.136	0.020	6.686	<.001	0.136	0.020	6.686	<.001

*Note:* ^∞^ = direct effect; ^+^ = indirect effect Step 2; ^*f*^ = indirect effect Step 1; SOMM = somato-motor mouth network.

**TableS17.** Mediation model parameters––Somatomotor-mouth network–amygdala connectivity in girls

	SOMM–left amygdala				SOMM–right amygdala			
	β	*S.E.*	β /*S.E*.	*p*	β	*S.E.*	β /*S.E.*	*p*
Family Environment^∞^	0.064	0.031	2.099	.036	0.026	0.035	0.0753	.452
Pubertal stage^+^	0.045	0.041	1.094	.274	−0. 005	0.037	−0.146	.884
Age	−0.004	0.031	−0.113	.910	0.010	0.035	0.281	.779
Race	−0.071	0.032	−2.208	.027	−0.052	0.032	−1.592	.111
	Outcome: Pubertal stage				Outcome: Pubertal stage			
Family Environment^*f*^	−0.171	0.034	−5.058	<.001	−0.171	0.034	−5.058	<.001
Age	0.300	0.030	9.991	.000	0.300	0.030	9.991	.000
Race	0.111	0.034	3.295	<.001	0.111	0.034	3.295	<.001

*Note:* ^∞^ = direct effect; ^+^ = indirect effect Step 2; ^*f*^ = indirect effect Step 1; SOMM = somato-motor mouth network.

**TableS20.** Mediation model parameters––Somatomotor-mouth network–amygdala connectivity in boys

	SOMM–left amygdala				SOMM–right amygdala			
	β	*S.E.*	β /*S.E*.	*p*	β	*S.E.*	β /*S.E.*	*p*
Family Environment^∞^	0.056	0.032	1.787	.074	−0.046	0.031	−1.474	.141
Pubertal stage^+^	−0.043	0.045	−0.972	.331	−0.023	0.044	−0.528	.597
Age	−0.031	0.031	−1.003	.316	−0.024	0.034	−0.698	.485
Race	0.034	0.029	1.160	.246	−0.002	0.031	−0.060	.952
	Outcome: Pubertal stage				Outcome: Pubertal stage			
Family Environment^*f*^	−0.142	0.036	−3.960	<.001	−0.142	0.036	−3.960	<.001
Age	0.223	0.035	6.289	<.001	0.223	0.035	6.289	<.001
Race	0.212	0.031	6.846	<.001	0.212	0.031	6.846	<.001

*Note:* ^∞^ = direct effect; ^+^ = indirect effect Step 2; ^*f*^ = indirect effect Step 1.SOMM = somato-motor mouth network.

**Table3.** Correlation between MRI measures

	ACC CA	ACC FA	Amygdala SV	CON–l amygdala FC	CON–r amygdala FC
ACC CT	−.082	−.072	.067	.003	.036
ACC CA		−.257	−.017	.033	.013
ACC FA			−.049	−.012	−.038
Amygdala SV				−.058	−.033
CON-– amygdala FC					.586

*Note:* All measures are residualized for data collection site. Gray matter measures were further residualized for total brain volume. ACC = anterior cingulate cortex; CT = cortical thickness; CA = cortical area; FA = fractional anisotropy; SC = subcortical volume; CON = cingulo-opercular network; l = left; r = right; FC = functional connectivity.

**TableS1.** Correlations among brain measures of motor processing

	Precentral CA	Precentral FA	SOMM–L Amygdala FC	SOMM-–R Amygdala FC
Precentral CT	−.423	.204	.070	.023
Precentral CA		−.114	−.046	−.011
Precentral FA			−.031	.033
SOMM–L Amygdala FC				−.197

*Note:* SOMM = somatomotor-mouth network; FC = functional connectivity; CT = cortical thickness; CA = cortical area; FA = fractional anisotropy.
