# Supplementary material for: Pubertal development mediates the association between family environment and brain structure and function in childhood
Source: Dev Psychopathol. 2020 May;32(2):687–702. doi: 10.1017/S0954579419000580 (PMC7525116; doi:10.1017/S0954579419000580)
Supplement: Supplementary file 1 [file S0954579419000580sup001.docx]

**Supplemental Materials**


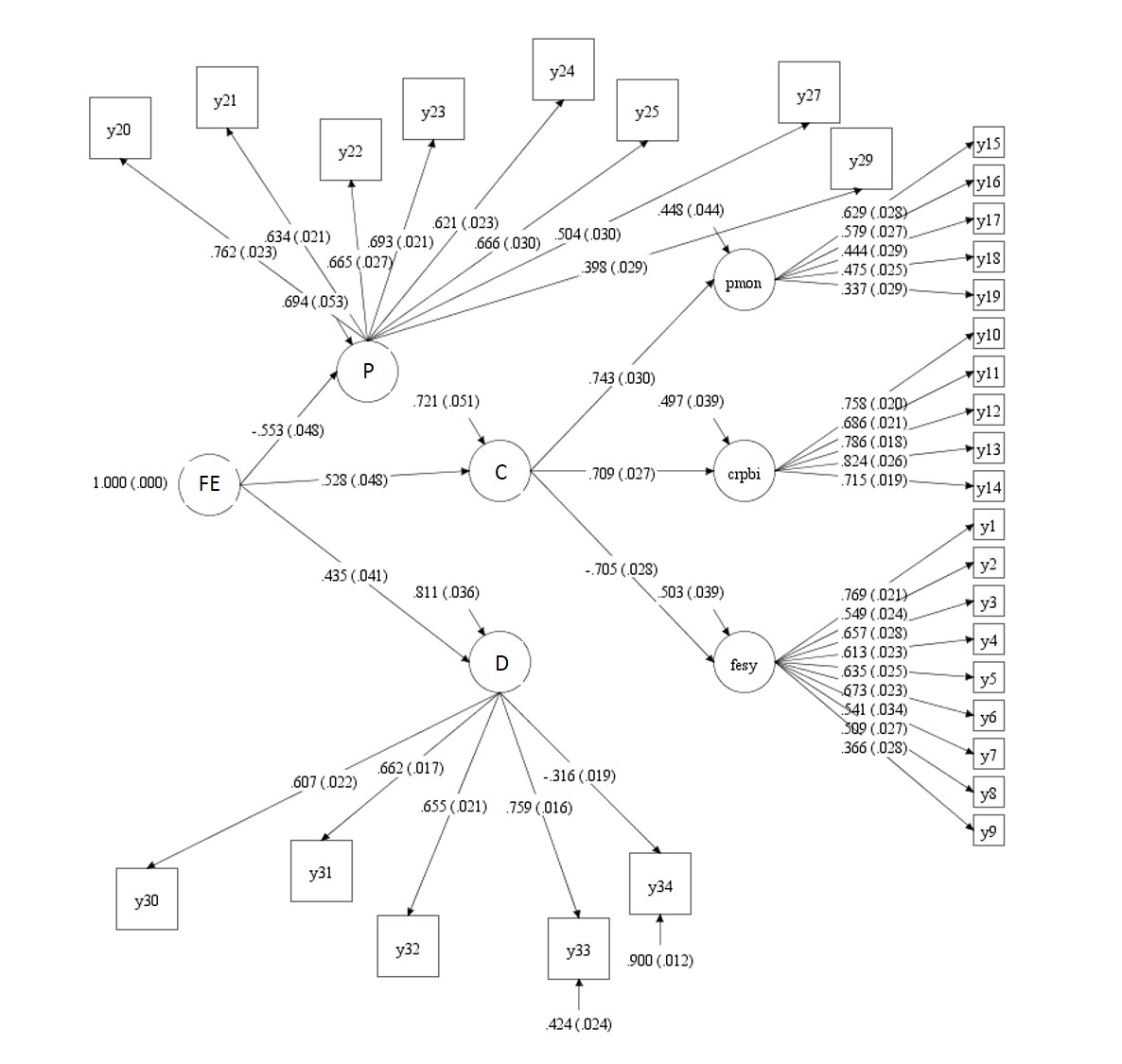


Figure S1. Structural Equation Model Family Environment. FE = Family Environment; P = Parent-reported latent variable; C = Child-report latent variable; D = Demographic and parental latent variable; pmon = Parental monitoring latent variable; crpbi = Child report of parent behavior inventory latent variable; fesy = Family Environment Scale youth report latent variable; y1-y9 = items of Conflict Scale of FESY; y10-y14 = Items of Parental acceptance scale of CRPBI; y15-y19 = items of Parental Monitory Scale; y20-y25, y27 = Items of Conflict scale of FES parent report; y29 = KSADS item on parent-child conflict; y30 = planned pregnancy; y31 = parental education; y32 = parental separation; y33 = family income; y34 = parental psychopathology

Table S1. Correlations among brain measures of motor processing

|  | Precentral CA | Precentral FA | SOMM-L Amygdala FC | SOMM- R Amygdala FC |
| --- | --- | --- | --- | --- |
| Precentral CT | -0.423 | .204 | .067 | -.034 |
| Precentral CA |  | -.114 | -.040 | -.008 |
| Precentral FA |  |  | -.034 | .040 |
| SOMM-L Amygdala FC |  |  |  | -.197 |

Note. SOMM = somatomotor-mouth network; FC = functional connectivity; CT = cortical thickness; CA = cortical area; FA = fractional anisotropy.


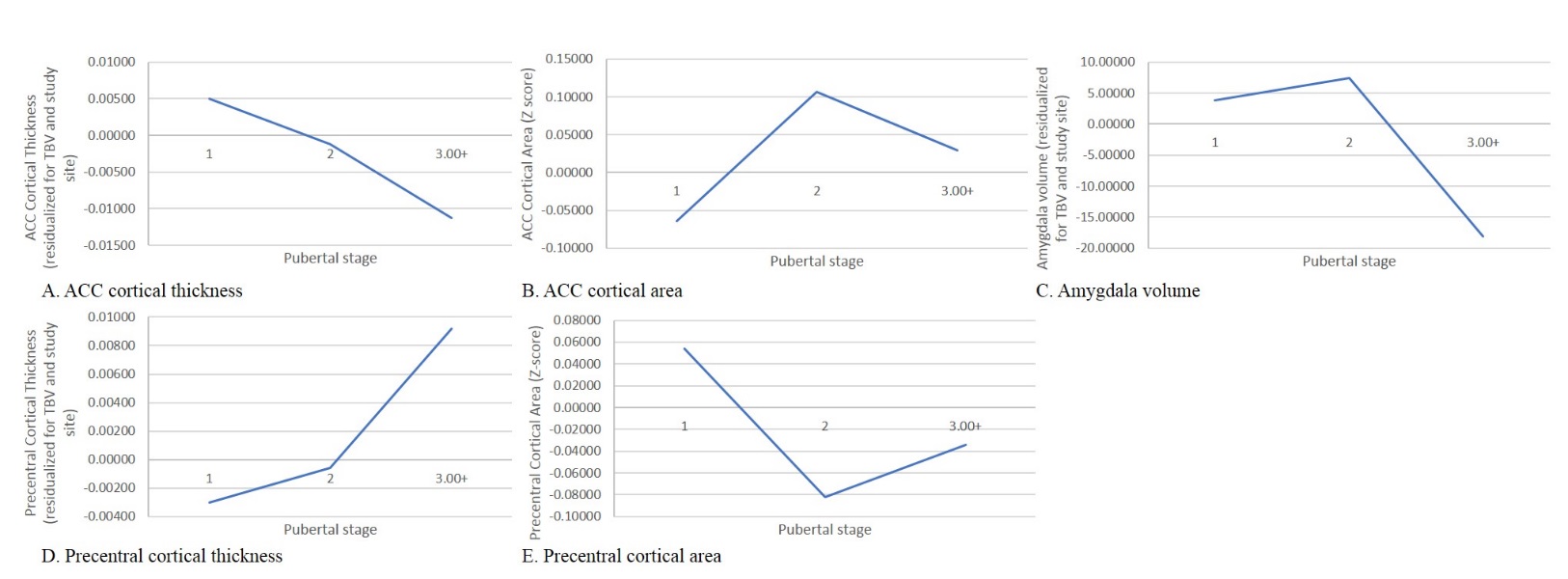


Figure S2. Gray matter by pubertal stage: A. anterior cingulate cortical thickness, B. anterior cingulate cortical area, C. amygdala volume, D. precentral cortical thickness, E. precentral cortical area. Note: ACC = anterior cingulate cortex; TBV = total brain volume


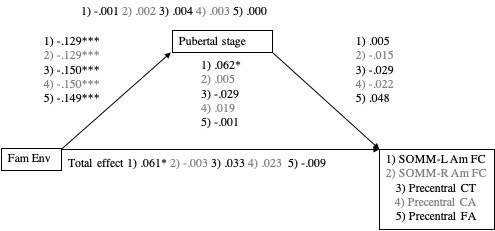


Figure S3. Mediation model of the association between Family Environment and motor processing measures. Values are standardized coefficients. * *p* < .01, ** *p* < .01, *** *p* < .001. Fam Env = Family environment; SOMM-Am FC = somatomotor-mouth network-amygdala functional connectivity; CT = cortical thickness; CA = cortical area; FA = fractional anisotropy.

Table S2. Quadratic mediation model of ACC CA and amygdala volume

|  | Model of ACC CA | | | |  | | | Amygdala volume | | | | | |
| --- | --- | --- | --- | --- | --- | --- | --- | --- | --- | --- | --- | --- | --- |
|  | *R* | *R^2^* | *F* | *p* | | *Df1* | *Df2* | *R* | *R^2^* | *F* | *p* | *Df1* | *Df2* |
|  | .113 | .013 | 3.998 | <.001 | | 8 | 2474 | 0.146 | 0.021 | 6.733 | <.001 | 8 | 2474 |
|  | *b* | *SE* | *t* | *p* | |  |  | *b* | *SE* | *t* | *p* |  |  |
| Constant | -0.850 | 0.354 | -2.400 | 0.017 | |  |  | -0.662 | 0.352 | -1.878 | .061 |  |  |
| Fam Env ^∞^ | -0.006 | 0.037 | -0.151 | .880 | |  |  | 0.044 | 0.037 | 1.209 | .227 |  |  |
| Pubertal stage | 0.384 | 0.144 | 2.663 | .008 | |  |  | 0.173 | 0.143 | 1.205 | .229 |  |  |
| Pubertal stage^2 +^ | -0.098 | 0.035 | -2.773 | .006 | |  |  | -0.039 | 0.035 | -1.109 | .268 |  |  |
| Age | 0.005 | 0.003 | 1.892 | .059 | |  |  | 0.003 | 0.003 | 1.087 | .277 |  |  |
| Sex | -0.191 | 0.046 | -4.148 | <.001 | |  |  | 0.266 | 0.046 | 5.785 | <.001 |  |  |
| Black | 0.096 | 0.080 | 1.201 | .230 | |  |  | -0.166 | 0.079 | -2.089 | .037 |  |  |
| Hispanic | -0.003 | 0.051 | -0.052 | .959 | |  |  | -0.074 | 0.050 | 1.471 | .141 |  |  |
| Other | 0.008 | 0.065 | 0.121 | .904 | |  |  | -0.025 | 0.065 | -0.381 | .704 |  |  |
| Model of pubertal stage | *R* | *Rsq* | *F* | *p* | | *Df1* | *Df2* | *R* | *Rsq* | *F* | *p* | *Df1* | *Df2* |
|  | 0.559 | 0.312 | 187.530 | <.001 | | 6 | 2476 | 0.559 | 0.312 | 187.530 | <.001 | 6 | 2476 |
|  | *b* | *SE* | *t* | *p* | |  |  | *b* | *SE* | *t* | *p* |  |  |
| Constant | -0.748 | 0.234 | -3.201 | 0.017 | |  |  | -0.748 | 0.234 | -3.201 | 0.014 |  |  |
| Fam Env^ƒ^ | -0.144 | 0.026 | -5.568 | <.001 | |  |  | -0.144 | 0.026 | -5.568 | <.001 |  |  |
| Age | 0.023 | 0.002 | 11.775 | <.001 | |  |  | 0.023 | 0.002 | 11.775 | <.001 |  |  |
| Sex | -0.798 | 0.028 | -28.149 | <.001 | |  |  | -0.799 | 0.028 | -28.149 | <.001 |  |  |
| Black | 0.633 | 0.055 | 11.541 | <.001 | |  |  | 0.633 | 0.055 | 11.541 | <.001 |  |  |
| Hispanic | 0.259 | 0.035 | 7.308 | <.001 | |  |  | 0.259 | 0.035 | 7.308 | <.001 |  |  |
| Other | 0.167 | 0.046 | 3.642 | <.001 | |  |  | 0.168 | 0.046 | 3.642 | <.001 |  |  |

Note. ^∞^ = direct effect; ^+^ = indirect effect step 2; ^ƒ^ = indirect effect step 1.

Text S1. Mediation models of motor processing areas

Gray matter

There were no significant total, direct or indirect effects of Family Environment on motor processing gray matter measures (CT: β = 0.033, *p* = 107, β = 0.029, *p* = .169, β = 0.004, *p* = 0.361. for total, direct and indirect effects, respectively; CA: β = 0.023, *p* = .289, β = ,0.019 *p* = .378, β = 0.003, *p* = .455 for total, direct and indirect effects, respectively), nor did we find evidence for surface area quadratic mediation.

None of the stratified precentral CT analyses provided significant results (precentral CT: girls: β = 0.011, *p* = .719, β = 0.007, *p* = .816, β = 0.004, *p* = .637 for total, direct and indirect effects, respectively; boys β = 0.036, *p* = .209, β = 0.033, *p* = .258, β = .003, *p* = .576 for total, direct and indirect effects, respectively.

For precentral CA, no significant associations were found for girls (girls: β = 0.018, *p* = .559, β = 0.015, *p* = .632, β = 0.003, *p* = .694 for total, direct and indirect linear effects, respectively). Nor did we find evidence for non-linear mediation. We found no evidence for linear or non-linear mediation in boys (β = 0.036, *p* = .235, β = 0.031, *p* = .318, β = 0.006, *p* = .347 for total, direct and indirect effects, respectively).

White matter

Neither in the total sample, nor in the stratified analyses did we find significant total, direct or indirect associations between Family Environment and precentral FA (total: β = -0.009, *p* = .703, β = -0.001, *p* = .950, β = -0.007, *p* = .155 for total, direct and indirect effects, respectively; girls: β = 0.003 *p* = .935, β = 0.007, *p* = .854, β = -0.004, *p* = .573 for total, direct and indirect effects, respectively; boys β = -0.018, *p* = .536, β = -0.005, *p* = .865, β = -0.013, *p* = .113 for total, direct and indirect effects, respectively).

Resting-state fMRI

For the resting-state model with motor processing measures only the total and direct effects of Family Environment on SOMM-left amygdala FC were significant (β = .061, *p* = .003, β = .062, *p* = .003, respectively), but not the indirect effect (β = -0.001, *p* = .894). No associations between Family Environment and SOMM-right amygdala were found (β = -0.003, *p* = .878, β = -0.005, *p* = .817, β = 0.002, *p* = .663, for total, direct, and indirect effects, respectively).

For the resting-state model with motor processing measures, in girls the total, and direct effects, and in boys only the total effect of Family Environment on SOMM- left amygdala FC were significant (girls: β = 0.093, *p* = .002, β = 0.083, *p* = .007, β = 0.010, *p* = .107 for total, direct and indirect effects, respectively; boys β = 0.056, *p* = .073, β = 0.050, *p* = .115, β = 0.006, *p* = .360. for total, direct and indirect effects, respectively). No significant associations were found between Family Environment and SOMM-right amygdala (girls: β = 0.060, *p* = .070, β = 0.055, *p* = .107, β = 0.005, *p* = .378 for total, direct and indirect effects, respectively; boys β = -0.026, *p* = .390, β = -0.032, *p* = .316, β = 0.005, *p* = .351 for total, direct and indirect effects, respectively).

Table S3. Mediation model parameters – precentral cortex cortical thickness and area

|  | Precental cortical thickness | | | | Precentral cortical area | | | |
| --- | --- | --- | --- | --- | --- | --- | --- | --- |
|  | β | *S.E.* | β/*S.E*. | *p* | β | *S.E.* | β /*S.E.* | *p* |
| Family Environment^∞^ | 0.029 | 0.021 | 1.376 | .169 | 0.019 | 0.022 | 0.882 | .378 |
| Pubertal stage^+^ | -0.029 | 0.031 | -0.929 | .353 | -0.022 | 0.029 | -0.766 | .443 |
| Age | -0.007 | 0.020 | -0.332 | .740 | 0.019 | 0.021 | 0.884 | .377 |
| Sex | -0.144 | 0.025 | -5.814 | .000 | 0.065 | 0.024 | 2.694 | .007 |
| Race | -0.025 | 0.021 | -1.184 | .236 | -0.001 | 0.021 | -0.060 | .952 |
|  | Outcome: pubertal stage | | | | Outcome: pubertal stage | | | |
| Family Environment^ƒ^ | -0.150 | 0.020 | -7.442 | <.001 | -0.150 | 0.020 | -7.442 | <.001 |
| Age | 0.218 | 0.019 | 11.392 | <.001 | 0.218 | 0.019 | 11.392 | <.001 |
| Sex | -0.511 | 0.015 | -33.433 | <.001 | -0.511 | 0.015 | -33.433 | <.001 |
| Race | 0.136 | 0.020 | 6.807 | <.001 | 0.136 | 0.020 | 6.806 | <.001 |

Note. ^∞^ = direct effect; ^+^ = indirect effect step 2; ^ƒ^ = indirect effect step 1.

Table S4. Quadratic mediation of precentral cortical area by pubertal stage

|  | *R* | *R^2^* | *F* | *p* | *Df1* | *Df2* |
| --- | --- | --- | --- | --- | --- | --- |
|  | .093 | .009 | 2.701 | .006 | 8 | 2474 |
|  | *b* | *SE* | *t* | *p* |  |  |
| Constant | -0.049 | 0.356 | -0.139 | .890 |  |  |
| Fam Env ^∞^ | 0.030 | 0.037 | 0.789 | .430 |  |  |
| Pubertal stage | -0.283 | 0.145 | -1.957 | .050 |  |  |
| Pubertal stage^2 +^ | 0.069 | 0.035 | 1.960 | .050 |  |  |
| Age | 0.002 | 0.003 | 0.629 | .529 |  |  |
| Sex | 0.150 | 0.046 | 3.244 | .001 |  |  |
| Black | -0.116 | 0.080 | -1.442 | .149 |  |  |
| Hispanic | -0.001 | 0.051 | -0.010 | .992 |  |  |
| Other | -0.007 | 0.046 | -0.103 | .918 |  |  |
| Model of pubertal stage | *R* | *R^2^* | *F* | *p* | *Df1* | *Df2* |
|  | 0.559 | 0.312 | 187.530 | <.001 | 6 | 2476 |
|  | *b* | *SE* | *t* | *p* |  |  |
| Constant | -0.748 | 0.233 | -3.201 | 0.014 |  |  |
| Fam Env^ƒ^ | -0.144 | 0.026 | -5.568 | <.001 |  |  |
| Age | 0.023 | 0.002 | 11.775 | <.001 |  |  |
| Sex | -0.799 | 0.028 | -28.149 | <.001 |  |  |
| Black | 0.633 | 0.055 | 11.541 | <.001 |  |  |
| Hispanic | 0.259 | 0.035 | 7.308 | <.001 |  |  |
| Other | 0.168 | 0.046 | 3.642 | <.001 |  |  |

Note. ^∞^ = direct effect; ^+^ = indirect effect step 2; ^ƒ^ = indirect effect step 1.

Table S5. Mediation model parameters – precentral cortex white matter fractional anisotropy

|  | β | *S.E.* | β/*S.E*. | *p* |
| --- | --- | --- | --- | --- |
| Family Environment^∞^ | -0.001 | 0.023 | -0.063 | .950 |
| Pubertal stage^+^ | 0.048 | 0.033 | 1.482 | .138 |
| Age | 0.081 | 0.022 | 3.721 | .000 |
| Sex | -0.053 | 0.026 | -2.035 | .042 |
| Race | 0.039 | 0.023 | 1.714 | .086 |
|  | Outcome: pubertal stage | | | |
| Family Environment^ƒ^ | -0.149 | 0.022 | -6.894 | <.001 |
| Age | 0.213 | 0.022 | 9.609 | <.001 |
| Sex | -0.507 | 0.017 | -29.495 | <.001 |
| Race | 0.141 | 0.021 | 6.695 | <.001 |

Note. ^∞^ = direct effect; ^+^ = indirect effect step 2; ^ƒ^ = indirect effect step 1.

Table S6. Mediation model parameters – somatomotor-mouth network-amygdala connectivity

|  | SOMM-left amygdala | | | | SOMM-right amygdala | | | |
| --- | --- | --- | --- | --- | --- | --- | --- | --- |
|  | β | *S.E.* | β/*S.E*. | *p* | β | *S.E.* | β /*S.E.* | *p* |
| Family Environment^∞^ | 0.062 | 0.021 | 2.946 | .003 | -0.005 | 0.023 | -0.232 | .817 |
| Pubertal stage^+^ | 0.005 | 0.033 | 0.135 | .892 | -0.015 | 0.033 | -0.444 | .657 |
| Age | -0.018 | 0.020 | -0.842 | .400 | -0.012 | 0.024 | -0.494 | .622 |
| Sex | 0.002 | 0.026 | 0.079 | .937 | 0.008 | 0.027 | 0.312 | .755 |
| Race | -0.015 | 0.021 | -0.708 | .479 | -0.016 | 0.021 | -0.759 | .448 |
|  | Outcome: pubertal stage | | | | Outcome: pubertal stage | | | |
| Family Environment^ƒ^ | -0.129 | 0.020 | -6.530 | <.001 | -0.129 | 0.020 | -6.530 | <.001 |
| Age | 0.231 | 0.020 | 11.483 | <.001 | 0.231 | 0.020 | 11.483 | <.001 |
| Sex | -0.508 | 0.015 | -32.913 | <.001 | -0.508 | 0.015 | -32.913 | <.001 |
| Race | 0.140 | 0.020 | 7.131 | <.001 | 0.140 | 0.020 | 7.131 | <.001 |

Note. ^∞^ = direct effect; ^+^ = indirect effect step 2; ^ƒ^ = indirect effect step 1; SOMM = somato-motor mouth network.

Table S7. Mediation model parameters – anterior cingulate cortical thickness and area and amygdala volume in girls

|  | ACC cortical thickness | | | | ACC cortical area | | | | Amygdala volume | | | |
| --- | --- | --- | --- | --- | --- | --- | --- | --- | --- | --- | --- | --- |
|  | β | *S.E.* | β/*S.E*. | *p* | β | *S.E.* | β /*S.E.* | *p* | β | *S.E.* | β /*S.E.* | *p* |
| Fam Env^∞^ | -0.026 | 0.031 | -0.838 | 0.402 | -0.028 | 0.030 | -0.919 | 0.358 | -0.034 | 0.056 | -0.603 | 0.546 |
| Pubertal stage^+^ | -0.100 | 0.037 | -2.714 | 0.007 | 0.000 | 0.039 | -0.003 | 0.998 | 0.048 | 0.089 | 0.536 | 0.592 |
| Age | -0.112 | 0.029 | -3.800 | 0.000 | 0.024 | 0.031 | 0.761 | 0.447 | -0.011 | 0.028 | -0.374 | 0.709 |
| Race | 0.004 | 0.031 | 0.125 | 0.901 | -0.002 | 0.029 | -0.068 | 0.945 | -0.018 | 0.062 | -0.297 | 0.767 |
|  | Outcome: pubertal stage | | | | Outcome: pubertal stage | | | | Outcome: pubertal stage | | | |
| Fam Env^ƒ^ | -0.198 | 0.031 | -6.492 | 0.000 | -0.198 | 0.031 | -6.492 | 0.000 | -0.211 | 0.033 | -6.357 | 0.000 |
| Age | 0.304 | 0.029 | 10.389 | 0.000 | 0.304 | 0.029 | 10.389 | 0.000 | 0.303 | 0.032 | 9.607 | 0.000 |
| Race | 0.118 | 0.032 | 3.743 | 0.000 | 0.118 | 0.032 | 3.743 | 0.000 | 0.19 | 0.035 | 3.448 | 0.001 |

Note. ^∞^ = direct effect; ^+^ = indirect effect step 2; ^ƒ^ = indirect effect step 1; ACC = anterior cingulate cortex

Table S8. Mediation model parameters – anterior cingulate white matter fractional anisotropy in girls

|  | β | *S.E.* | β/*S.E*. | *p* |
| --- | --- | --- | --- | --- |
| Family Environment^∞^ | -0.038 | 0.037 | -1.042 | 0.298 |
| Pubertal stage^+^ | 0.129 | 0.039 | 3.331 | 0.001 |
| Age | 0.019 | 0.034 | 0.573 | 0.567 |
| Race | 0.026 | 0.034 | 0.761 | 0.447 |
|  | Outcome: pubertal stage | | | |
| Family Environment^ƒ^ | -0.156 | 0.033 | -4.721 | 0.000 |
| Age | 0.292 | 0.033 | 8.893 | 0.000 |
| Race | 0.126 | 0.034 | 3.738 | 0.000 |

Note. ^∞^ = direct effect; ^+^ = indirect effect step 2; ^ƒ^ = indirect effect step 1.

Table S9. Mediation model parameters – cinculo-opercular network-amygdala connectivity in girls

|  | CON-left amygdala | | | | CON-right amygdala | | | |
| --- | --- | --- | --- | --- | --- | --- | --- | --- |
|  | β | *S.E.* | β/*S.E*. | *p* | β | *S.E.* | β /*S.E.* | *p* |
| Family Environment^∞^ | 0.090 | 0.031 | 2.904 | .004 | 0.053 | 0.034 | 1.551 | .121 |
| Pubertal stage^+^ | -0.070 | 0.035 | -2.004 | .045 | -0.039 | 0.036 | -1.068 | .286 |
| Age | -0.014 | 0.032 | -0.0440 | .660 | 0.020 | 0.030 | 0.645 | 0.519 |
| Race | -0.051 | 0.032 | -1.607 | .108 | -0.057 | 0.031 | -1.866 | .062 |
|  | Outcome: pubertal stage | | | | Outcome: pubertal stage | | | |
| Family Environment^ƒ^ | -0.158 | 0.032 | -4.985 | 0.000 | -0.158 | 0.032 | -4.985 | 0.000 |
| Age | 0.305 | 0.029 | 10.392 | 0.000 | 0.305 | 0.029 | 10.392 | 0.000 |
| Race | 0.115 | 0.032 | 3.573 | 0.000 | 0.115 | 0.032 | 3.573 | 0.000 |

Note. ^∞^ = direct effect; ^+^ = indirect effect step 2; ^ƒ^ = indirect effect step 1; CON = cingulo-opercular network

Table S10. Mediation model parameters – anterior cingulate cortical thickness and area and amygdala volume in boys

|  | ACC cortical thickness | | | | ACC cortical area | | | | Amygdala volume | | | |
| --- | --- | --- | --- | --- | --- | --- | --- | --- | --- | --- | --- | --- |
|  | β | *S.E.* | β/*S.E*. | *p* | β | *S.E.* | β /*S.E.* | *p* | β | *S.E.* | β /*S.E.* | *p* |
| Fam Env^∞^ | 0.018 | 0.029 | 0.623 | 0.533 | 0.001 | 0.031 | 0.037 | 0.970 | 0.077 | 0.046 | 1.661 | 0.097 |
| Pubertal stage^+^ | -0.054 | 0.037 | -1.433 | 0.152 | 0.012 | 0.036 | 0.327 | 0.744 | -0.042 | 0.090 | -0.470 | 0.638 |
| Age | -0.101 | 0.029 | -3.538 | 0.262 | 0.043 | 0.029 | 1.456 | 0.143 | 0.014 | 0.018 | 0.754 | 0.451 |
| Race | -0.034 | 0.031 | -1.121 | 0.000 | 0.009 | 0.07 | 0.328 | 0.743 | 0.033 | 0.047 | 0.690 | 0.490 |
|  | Outcome: pubertal stage | | | | Outcome: pubertal stage | | | | Outcome: pubertal stage | | | |
| Fam Env^ƒ^ | -0.141 | 0.034 | -4.102 | 0.000 | -0.141 | 0.034 | -4.102 | 0.000 | -0.108 | 0.035 | -3.108 | 0.002 |
| Age | 0.182 | 0.034 | 5.354 | 0.000 | 0.182 | 0.034 | 5.354 | 0.000 | 0.193 | 0.037 | 5.200 | 0.000 |
| Race | 0.202 | 0.031 | 6.487 | 0.000 | 0.202 | 0.031 | 6.487 | 0.000 | 0.214 | 0.032 | 6.687 | 0.000 |

Note. ^∞^ = direct effect; ^+^ = indirect effect step 2; ^ƒ^ = indirect effect step 1; ACC = anterior cingulate cortex

Table S11. Mediation model parameters – anterior cingulate white matter fractional anisotropy in boys

|  | β | *S.E.* | β/*S.E*. | *p* |
| --- | --- | --- | --- | --- |
| Family Environment^∞^ | -0.077 | 0.028 | -2.721 | 0.007 |
| Pubertal stage^+^ | -0.007 | 0.042 | -0.167 | 0.867 |
| Age | 0.030 | 0.031 | 0.963 | 0.085 |
| Race | 0.057 | 0.033 | 1.724 | 0.336 |
|  | Outcome: pubertal stage | | | |
| Family Environment^ƒ^ | -0.185 | 0.035 | -5.352 | 0.000 |
| Age | 0.208 | 0.036 | 6.058 | 0.000 |
| Race | 0.183 | 0.034 | 5.044 | 0.000 |

Note. ^∞^ = direct effect; ^+^ = indirect effect step 2; ^ƒ^ = indirect effect step 1.

Table S12. Mediation model parameters – cinculo-opercular network-amygdala connectivity in boys

|  | CON-left amygdala | | | | CON-right amygdala | | | |
| --- | --- | --- | --- | --- | --- | --- | --- | --- |
|  | β | *S.E.* | β/*S.E*. | *p* | β | *S.E.* | β /*S.E.* | *p* |
| Family Environment^∞^ | 0.039 | 0.030 | 1.286 | 0.198 | 0.011 | 0.032 | 0.334 | 0.738 |
| Pubertal stage^+^ | -0.038 | 0.041 | -0.940 | 0.347 | -0.064 | 0.040 | -1.596 | 0.111 |
| Age | 0.042 | 0.031 | 1.375 | 0.169 | 0.041 | 0.029 | -0.410 | 0.682 |
| Race | -0.029 | 0.029 | -1.017 | 0.309 | -0.012 | 0.030 | 1.363 | 0.173 |
|  | Outcome: pubertal stage | | | | Outcome: pubertal stage | | | |
| Family Environment^ƒ^ | -0.140 | 0.033 | -4.202 | 0.000 | -0.140 | 0.033 | -4.202 | 0.000 |
| Age | 0.217 | 0.035 | 6.246 | 0.000 | 0.217 | 0.035 | 6.246 | 0.000 |
| Race | 0.218 | 0.030 | 7.246 | 0.000 | 0.218 | 0.030 | 7.246 | 0.000 |

Note. ^∞^ = direct effect; ^+^ = indirect effect step 2; ^ƒ^ = indirect effect step 1; CON = cingulo-opercular network

Table S13. Quadratic mediation of ACC CA in boys and girls separately

| ACC CA | Girls | | | | | | Boys | | | | | |
| --- | --- | --- | --- | --- | --- | --- | --- | --- | --- | --- | --- | --- |
|  | *R* | *R^2^* | *F* | *p* | *Df1* | *Df2* | *R* | *R^2^* | *F* | *p* | *Df1* | *Df2* |
|  | .093 | .009 | 1.433 | .188 | 7 | 1160 | .067 | .005 | 0.852 | .544 | 7 | 1307 |
|  | *b* | *SE* | *t* | *p* |  |  | *b* | *SE* | *t* | *p* |  |  |
| Constant | -0.865 | 0.521 | -1.660 | .097 |  |  | -0.940 | 0.493 | -1.908 | .057 |  |  |
| Fam Env^∞^ | -0.036 | 0.054 | -0.678 | .498 |  |  | 0.017 | 0.051 | 0.331 | .741 |  |  |
| Pubertal stage | 0.525 | 0.193 | 2.717 | .007 |  |  | 0.123 | 0.242 | 0.509 | .611 |  |  |
| Pubertal stage^2 +^ | -0.131 | 0.045 | -2.869 | .004 |  |  | -0.027 | 0.065 | -0.418 | .676 |  |  |
| Age | 0.005 | 0.004 | 1.116 | .265 |  |  | 0.006 | 0.004 | 1.554 | .120 |  |  |
| Black | 0.022 | 0.116 | 0.190 | .849 |  |  | 0.157 | 0.111 | 1.416 | .157 |  |  |
| Hispanic | -0.061 | 0.073 | -.844 | .399 |  |  | 0.044 | 0.071 | 0.625 | .532 |  |  |
| Other | -0.046 | 0.090 | -0.509 | .611 |  |  | 0.060 | 0.095 | 0.629 | .530 |  |  |
| Model of pubertal stage | *R* | *R^2^* | *F* | *P* | *Df1* | *Df2* | *R* | *R^2^* | *F* | *p* | *Df1* | *Df2* |
|  | .409 | .167 | 46.601 | <.001 | 5 | 1162 | .328 | .108 | 31.655 | <.001 | 5 | 1309 |
|  | *b* | *SE* | *t* | *P* |  |  | *b* | *SE* | *t* | *p* |  |  |
| Constant | -2.359 | 0.403 | -5.855 | <.001 |  |  | -0.128 | 0.254 | -0.507 | .613 |  |  |
| Fam Env^ƒ^ | -0.214 | 0.045 | -4.771 | <.001 |  |  | -0.073 | 0.028 | -2.627 | .009 |  |  |
| Age | 0.036 | 0.003 | 10.803 | <.001 |  |  | 0.011 | 0.002 | 5.326 | <.001 |  |  |
| Black | 0.756 | 0.095 | 7.947 | <.001 |  |  | 0.535 | 0.059 | 9.049 | <.001 |  |  |
| Hispanic | 0.261 | 0.061 | 4.289 | <.001 |  |  | 0.253 | 0.039 | 6.556 | <.001 |  |  |
| Other | 0.153 | 0.076 | 2.010 | .045 |  |  | 0.174 | 0.052 | 3.349 | .001 |  |  |

Note. ^∞^ = direct effect; ^+^ = indirect effect step 2; ^ƒ^ = indirect effect step 1; ACC = anterior cingulate cortex

Table S14. Quadratic mediation of amygdala volume in boys and girls separately

| Amygdala volume | Girls | | | | | | Boys | | | | | |
| --- | --- | --- | --- | --- | --- | --- | --- | --- | --- | --- | --- | --- |
|  | *R* | *R^2^* | *F* | *p* | *Df1* | *Df2* | *R* | *R^2^* | *F* | *p* | *Df1* | *Df2* |
|  | .065 | .004 | 0.703 | .670 | 7 | 1160 | .120 | .014 | 2.715 | .009 | 7 | 1307 |
|  | *b* | *SE* | *t* | *p* |  |  | *b* | *SE* | *t* | *p* |  |  |
| Constant | -0.180 | 0.491 | -0.367 | .714 |  |  | -0.819 | 0.510 | -1.606 | 0.109 |  |  |
| Fam Env^∞^ | -0.040 | 0.051 | -0.792 | .429 |  |  | 0.109 | 0.052 | 2.09 | 0.037 |  |  |
| Pubertal stage | 0.251 | 0.182 | 1.382 | .167 |  |  | 0.173 | 0.250 | 0.692 | 0.489 |  |  |
| Pubertal stage^2 +^ | -0.053 | 0.043 | -1.241 | .215 |  |  | -0.048 | 0.067 | -0.721 | 0.471 |  |  |
| Age | -0.002 | 0.004 | -0.370 | .711 |  |  | 0.007 | 0.004 | 1.644 | 0.100 |  |  |
| Black | -0.146 | 0.109 | -1.333 | .183 |  |  | -0.179 | 0.115 | -1.566 | 0.118 |  |  |
| Hispanic | -0.038 | 0.068 | -0.551 | .582 |  |  | 0.172 | 0.074 | 2.34 | 0.020 |  |  |
| Other | -0.086 | 0.085 | -1.027 | .311 |  |  | 0.033 | 0.099 | 0.336 | 0.737 |  |  |
| Model of pubertal stage | *R* | *R^2^* | *F* | *p* | *Df1* | *Df2* | *R* | *R^2^* | *F* | *p* | *Df1* | *Df2* |
|  | .409 | .167 | 46.601 | <.001 | 5 | 1162 | .328 | .108 | 31.655 | <.001 | 5 | 1309 |
|  | *b* | *SE* | *t* | *P* |  |  | *b* | *SE* | *t* | *p* |  |  |
| Constant | -2.359 | 0.403 | -5.855 | <.001 |  |  | -0.128 | 0.254 | -0.507 | 0.613 |  |  |
| Fam Env^ƒ^ | -0.214 | 0.045 | -4.771 | <.001 |  |  | -0.073 | 0.028 | -2.627 | .009 |  |  |
| Age | 0.036 | 0.003 | 10.803 | <.001 |  |  | 0.011 | 0.002 | 5.326 | <.001 |  |  |
| Black | 0.756 | 0.095 | 7.947 | <.001 |  |  | 0.535 | 0.059 | 9.049 | <.001 |  |  |
| Hispanic | 0.261 | 0.061 | 4.289 | <.001 |  |  | 0.253 | 0.039 | 6.556 | <.001 |  |  |
| Other | 0.153 | 0.076 | 2.010 | .045 |  |  | 0.174 | 0.052 | 3.45 | .001 |  |  |

Note. ^∞^ = direct effect; ^+^ = indirect effect step 2; ^ƒ^ = indirect effect step 1.

Table S15. Mediation model parameters – precentral cortex cortical thickness and area in girls

|  | Precental cortical thickness | | | | Precentral cortical area | | | |
| --- | --- | --- | --- | --- | --- | --- | --- | --- |
|  | β | *S.E.* | β/*S.E*. | *p* | β | *S.E.* | β /*S.E.* | *p* |
| Family Environment^∞^ | 0.007 | 0.032 | 0.233 | 0.816 | 0.015 | 0.031 | 0.479 | 0.632 |
| Pubertal stage^+^ | -0.019 | 0.039 | -0.478 | 0.633 | -0.015 | 0.037 | -0.398 | 0.691 |
| Age | -0.024 | 0.029 | -0.804 | 0.421 | 0.040 | 0.031 | 1.315 | 0.189 |
| Race | -0.048 | 0.031 | -1.528 | 0.127 | 0.010 | 0.033 | 0.293 | 0.769 |
|  | Outcome: pubertal stage | | | | Outcome: pubertal stage | | | |
| Family Environment^ƒ^ | -0.198 | 0.031 | -6.492 | 0.000 | -0.198 | 0.031 | -6.492 | 0.000 |
| Age | 0.304 | 0.029 | 10.389 | 0.000 | 0.304 | 0.029 | 10.389 | 0.000 |
| Race | 0.118 | 0.032 | 3.743 | 0.000 | 0.118 | 0.032 | 3.743 | 0.000 |

Note. ^∞^ = direct effect; ^+^ = indirect effect step 2; ^ƒ^ = indirect effect step 1.

Table S16. Mediation model parameters – precentral cortex white matter fractional anisotropy in girls

|  | β | *S.E.* | β/*S.E*. | *p* |
| --- | --- | --- | --- | --- |
| Family Environment^∞^ | 0.007 | 0.036 | 0.184 | 0.854 |
| Pubertal stage^+^ | 0.023 | 0.040 | 0.580 | 0.562 |
| Age | 0.078 | 0.033 | 2.400 | 0.016 |
| Race | 0.023 | 0.033 | 0.689 | 0.491 |
|  | Outcome: pubertal stage | | | |
| Family Environment^ƒ^ | -0.156 | 0.033 | -4.271 | 0.000 |
| Age | 0.292 | 0.033 | 8.893 | 0.000 |
| Race | 0.126 | 0.034 | 3.738 | 0.000 |

Note. ^∞^ = direct effect; ^+^ = indirect effect step 2; ^ƒ^ = indirect effect step 1.

Table S17. Mediation model parameters – somatomotor-mouth network-amygdala connectivity in girls

|  | SOMM-left amygdala | | | | SOMM-right amygdala | | | |
| --- | --- | --- | --- | --- | --- | --- | --- | --- |
|  | β | *S.E.* | β/*S.E*. | *p* | β | *S.E.* | β /*S.E.* | *p* |
| Family Environment^∞^ | 0.083 | 0.031 | 2.683 | .007 | 0.055 | 0.034 | 1.612 | .107 |
| Pubertal stage^+^ | -0.060 | 0.035 | -1.719 | .086 | -0. 033 | 0.036 | -0.916 | .360 |
| Age | -0.020 | 0.032 | -0.646 | .518 | 0.013 | 0.030 | 0.441 | .659 |
| Race | -0.039 | 0.032 | -1.215 | .224 | -0.054 | 0.031 | -1.764 | .078 |
|  | Outcome: pubertal stage | | | | Outcome: pubertal stage | | | |
| Family Environment^ƒ^ | -0.158 | 0.032 | -4.985 | .000 | -0.158 | 0.032 | -4.985 | .000 |
| Age | 0.305 | 0.029 | 10.392 | .000 | 0.305 | 0.029 | 10.392 | .000 |
| Race | 0.115 | 0.032 | 3.573 | .000 | 0.115 | 0.032 | 3.573 | .000 |

Note. ^∞^ = direct effect; ^+^ = indirect effect step 2; ^ƒ^ = indirect effect step 1; SOMM = somato-motor mouth network.

Table S18. Mediation model parameters – precentral cortex cortical thickness and area in boys

|  | Precental cortical thickness | | | | Precentral cortical area | | | |
| --- | --- | --- | --- | --- | --- | --- | --- | --- |
|  | β | *S.E.* | β/*S.E*. | *p* | β | *S.E.* | β /*S.E.* | *p* |
| Family Environment^∞^ | 0.033 | 0.029 | 1.132 | -.258 | 0.031 | 0.031 | -.998 | 0.318 |
| Pubertal stage^+^ | -0.021 | 0.037 | -0.577 | 0.564 | -0.040 | 0.039 | -1.006 | 0.314 |
| Age | 0.006 | 0.028 | 0.152 | 0.879 | 0.005 | 0.028 | 0.194 | 0.662 |
| Race | 0.004 | 0.031 | 0.193 | 0.847 | -0.014 | 0.032 | -0.437 | 0.846 |
|  | Outcome: pubertal stage | | | | Outcome: pubertal stage | | | |
| Family Environment^ƒ^ | -0.141 | 0.034 | -4.102 | 0.000 | -0.141 | 0.034 | -4.102 | 0.000 |
| Age | 0.182 | 0.034 | 5.354 | 0.000 | 0.182 | 0.034 | 5.354 | 0.000 |
| Race | 0.202 | 0.031 | 6.487 | 0.000 | 0.202 | 0.031 | 6.487 | 0.000 |

Note. ^∞^ = direct effect; ^+^ = indirect effect step 2; ^ƒ^ = indirect effect step 1.

Table S19. Mediation model parameters – precentral cortex white matter fractional anisotropy in boys

|  | β | *S.E.* | β/*S.E*. | *p* |
| --- | --- | --- | --- | --- |
| Family Environment^∞^ | -0.005 | 0.029 | -0.170 | 0.865 |
| Pubertal stage^+^ | 0.069 | 0.042 | 1.655 | 0.098 |
| Age | 0.083 | 0.030 | 2.768 | 0.006 |
| Race | 0.050 | 0.031 | 1.584 | 0.113 |
|  | Outcome: pubertal stage | | | |
| Family Environment^ƒ^ | -0.185 | 0.035 | -5.352 | 0.000 |
| Age | 0.208 | 0.036 | 6.058 | 0.000 |
| Race | 0.183 | 0.034 | 5.044 | 0.000 |

Note. ^∞^ = direct effect; ^+^ = indirect effect step 2; ^ƒ^ = indirect effect step 1.

Table S20. Mediation model parameters – somatomotor-mouth network-amygdala connectivity in boys

|  | SOMM-left amygdala | | | | SOMM-right amygdala | | | |
| --- | --- | --- | --- | --- | --- | --- | --- | --- |
|  | β | *S.E.* | β/*S.E*. | *p* | β | *S.E.* | β /*S.E.* | *p* |
| Family Environment^∞^ | 0.050 | 0.032 | 1.576 | 0.115 | -0.032 | 0.032 | -1.003 | 0.316 |
| Pubertal stage^+^ | -0.041 | 0.042 | -0.990 | 0.322 | -0.038 | 0.040 | -0.960 | 0.337 |
| Age | -0.033 | 0.029 | -1.114 | 0.265 | -0.015 | 0.033 | -0.453 | 0.651 |
| Race | 0.025 | 0.028 | 0.878 | 0.380 | 0.017 | 0.029 | 0.584 | 0.559 |
|  | Outcome: pubertal stage | | | | Outcome: pubertal stage | | | |
| Family Environment^ƒ^ | -0.140 | 0.033 | -4.202 | 0.000 | -0.140 | 0.033 | -4.202 | 0.000 |
| Age | 0.217 | 0.035 | 6.297 | 0.000 | 0.217 | 0.035 | 6.297 | 0.000 |
| Race | 0.218 | 0.030 | 7.246 | 0.000 | 0.218 | 0.030 | 7.246 | 0.000 |

Note. ^∞^ = direct effect; ^+^ = indirect effect step 2; ^ƒ^ = indirect effect step 1. SOMM = somato-motor mouth network.

Table S21. Quadratic mediation of precentral CA in boys and girls separately

| Precentral CA | Girls | | | | | | Boys | | | | | |
| --- | --- | --- | --- | --- | --- | --- | --- | --- | --- | --- | --- | --- |
|  | *R* | *R^2^* | *F* | *p* | *Df1* | *Df2* | *R* | *R^2^* | *F* | *p* | *Df1* | *Df2* |
|  | .058 | .003 | 0.554 | .794 | 7 | 1160 | .088 | .008 | 1.467 | 0.175 | 7 | 1307 |
|  | *b* | *SE* | *t* | *p* |  |  | *b* | *SE* | *t* | *p* |  |  |
| Constant | -0.270 | 0.515 | -0.526 | .599 |  |  | 0.252 | 0.501 | 0.502 | .615 |  |  |
| Fam Env^∞^ | 0.004 | 0.053 | 0.077 | .939 |  |  | 0.049 | 0.051 | 0.953 | .341 |  |  |
| Pubertal stage | -0.197 | 0.191 | -1.030 | .303 |  |  | -0.334 | 0.246 | -1.359 | .174 |  |  |
| Pubertal stage^2 +^ | 0.048 | 0.045 | 1.057 | .291 |  |  | 0.082 | 0.066 | 1.249 | .217 |  |  |
| Age | 0.003 | 0.004 | 0.728 | .466 |  |  | 0.001 | 0.004 | 0.238 | .812 |  |  |
| Black | 0.007 | 0.115 | 0.058 | .954 |  |  | -0.211 | 0.113 | -1.876 | .061 |  |  |
| Hispanic | -0.055 | 0.072 | -0.774 | .439 |  |  | 0.049 | 0.073 | 0.684 | .494 |  |  |
| Other | 0.081 | 0.089 | 0.911 | .363 |  |  | -0.010 | 0.096 | -1.035 | .301 |  |  |
| Model of pubertal stage | *R* | *R^2^* | *F* | *p* | *Df1* | *Df2* | *R* | *R^2^* | *F* | *p* | *Df1* | *Df2* |
|  | .409 | .167 | 46.601 | <.001 | 5 | 1162 | .328 | .108 | 31.656 | <.001 | 5 | 1309 |
|  | *b* | *SE* | *t* | *P* |  |  | *b* | *SE* | *t* | *p* |  |  |
| Constant | -2.359 | 0.403 | -5.855 | <.001 |  |  | -0.128 | 0.254 | -0.507 | 0.613 |  |  |
| Fam Env^ƒ^ | -0.214 | 0.045 | -4.771 | <.001 |  |  | -0.073 | 0.028 | -2.627 | .009 |  |  |
| Age | 0.036 | 0.003 | 10.803 | <.001 |  |  | 0.011 | 0.002 | 5.326 | <.001 |  |  |
| Black | 0.756 | 0.095 | 7.947 | <.001 |  |  | 0.535 | 0.059 | 9.049 | <.001 |  |  |
| Hispanic | 0.261 | 0.061 | 4.289 | <.001 |  |  | 0.253 | 0.039 | 6.556 | <.001 |  |  |
| Other | 0.153 | 0.076 | 2.010 | .045 |  |  | 0.174 | 0.052 | 3.35 | .001 |  |  |

Note. ^∞^ = direct effect; ^+^ = indirect effect step 2; ^ƒ^ = indirect effect step 1.
